# The microtubule-binding protein EML3 is required for mammalian embryonic growth and cerebral cortical development, and Eml3 null mice are a model of cobblestone brain malformation

**DOI:** 10.7554/eLife.107102

**Published:** 2026-07-09

**Authors:** Isabelle Carrier, Eduardo Diez, Valerio E Piscopo, Susanne Bechstedt, Hans van Bokhoven, Myriam Srour, Albert M Berghuis, Stefano Stifani, Yojiro Yamanaka, Roderick McInnes

**Affiliations:** 1 Lady Davis Research Institute for Medical Research of the Montreal Jewish General Hospital, McGill University Montréal Canada; 2 Montreal Neurological Institute-Hospital, McGill University Montréal Canada; 3 Department of Anatomy and Cell Biology, McGill University Montréal Canada; 4 Centre de recherche en biologie structurale, McGill University Montréal Canada; 5 Department of Human Genetics, Radboud University Medical Center, Donders Institute for Brain, Cognition, and Behaviour Nijmegen Netherlands; 6 Montreal Children’s Hospital, MUHC-RI Montréal Canada; 7 Department of Pediatrics, McGill University Montréal Canada; 8 Department of Neurology and Neurosurgery, McGill University Montréal Canada; 9 Department of Biochemistry, McGill University Montréal Canada; 10 Department of Microbiology and Immunology, McGill University Montréal Canada; 11 Goodman Cancer Institute, McGill University Montréal Canada; 12 Department of Human Genetics, McGill University Montréal Canada; Universidad Nacional Autónoma de México Mexico; Universitat de Barcelona Spain

**Keywords:** cortical malformation, pial basement membrane, developmental delay, perinatal lethality, Mouse

## Abstract

The cerebral cortex is a multi-layered structure generated through the migration of neural precursors from their birthplace in the ventricular zone to their destination within the cortical plate. Neuronal migration defects are responsible for many human pathologies collectively called neuronal migration disorders, which include subcortical band heterotopia and cobblestone brain (COB) malformation. One example of a protein involved in a neuronal migration disorder is the echinoderm microtubule-associated protein-like 1 (EML1) protein, one of six members of the mammalian EML family. Absence of EML1 protein results in subcortical band heterotopia in mice and humans. Here, we report that the absence of the paralogous protein EML3 leads to delayed embryonic development and small size, and a COB-like phenotype with neuronal ectopias in the dorsal telencephalon. We found that EML3 is expressed in the neuroepithelium and meningeal mesenchyme when those tissues participate in pial basement membrane (PBM) formation. Transmission electron microscopy demonstrated that the extracellular matrix of the PBM is structurally abnormal in *Eml3* null mice when the first radially migrating neurons arrive. The reduced structural integrity of the PBM leads to focal over-migration of neurons into the subarachnoid space. These findings strengthen the link between the EML protein family and cortical neuronal migration defects by identifying *Eml3* as the first EML family member whose absence leads to over-migration of neuroblasts. Moreover, we report the first COB-like phenotype with PBM structural defects when a single microtubule-associated protein is deleted.

## Introduction

The cerebral cortex is generated through a series of steps that include the migration of neural precursors (neuroblasts) from their birthplace in the ventricular zone to their destination in the cortical plate (Gupta et al., 2002; Noctor et al., 2008). Neural precursors arise from progenitor cells called radial glia that are themselves differentiated from neuroepithelial cells beginning at embryonic day 10.5 (E10.5) in the mouse (Götz and Huttner, 2005; Noctor et al., 2008). Both neuroepithelial cells and radial glia are polarized cells that span the entire developing cerebral cortex (Götz and Huttner, 2005; Noctor et al., 2008). Neuroepithelial cells have a strictly proliferative (symmetric) cell division mode that yields two identical daughter cells (Kriegstein and Alvarez-Buylla, 2009; Taverna and Huttner, 2010). Radial glia, in contrast, switch to a neurogenic (asymmetric) mode of cell division that yields one radial glia and a neuroblast committed to becoming a neuron during neurogenesis (Noctor et al., 2008; Taverna and Huttner, 2010). The mammalian cortex develops in an inside-out manner, with radially migrating neuroblasts traversing earlier-formed layers to reach the cortical plate’s outer surface (Noctor et al., 2004; Valiente and Marín, 2010). Each layer emerges with precise timing, regulated by defined molecular and transcriptional cues (Bayatti et al., 2008; Bedogni et al., 2010; Hoerder-Suabedissen and Molnár, 2015). Radial glial fibers guide the neuroblasts along the radial axis. Thus, radial glia serve as both neural progenitors and scaffolds for migrating neurons (Anthony et al., 2004; Götz and Huttner, 2005). In mice, neuroblast radial migration starts just before E11.5 and is completed by E16.5 (Kriegstein et al., 2006; Miyata et al., 2004).

There are physical boundaries as well as molecular stop signals that define when a migrating neuroblast has reached its destination (Kawauchi, 2012). The pial basement membrane (PBM) serves both as a physical barrier and as a source of molecular cues (Siegenthaler and Pleasure, 2011). The PBM is made up of an extracellular matrix (ECM) and the basal endfeet of neuroepithelial cells or radial glia depending on the embryonic developmental stage (Belvindrah et al., 2011; Götz and Huttner, 2005). The ECM components are secreted from the neural precursors as well as from the overlaying mesenchymal cells (Franco and Müller, 2011). Several ECM components have self-assembling properties and also interact with cell surface receptors on the basal endfeet of neural precursors, thus anchoring and stabilizing the ECM (Chiquet-Ehrismann and Tucker, 2011; Yurchenco, 2015). Once the ECM is synthesized and deposited, it undergoes constant remodeling (Bonnans et al., 2014; Maeda, 2015).

Cortical neuronal migration defects cause alterations in the structure of the neocortex and are frequently associated with severe epilepsy (Barkovich et al., 2012; Guerrini and Dobyns, 2014). These disorders include lissencephaly, pachygyria, polymicrogyria (PMG), periventricular nodular heterotopia, subcortical band heterotopia, and cobblestone brain (COB) malformation (Barkovich et al., 2012; Guerrini and Filippi, 2005). COB malformation is a neuronal over-migration condition characterized by an irregular brain surface with nodular protrusions into the overlaying subarachnoid space (Dobyns and Truwit, 1995; Guerrini and Dobyns, 2014). In contrast, subcortical band heterotopia is due to a failure of neurons to migrate to the cerebral cortex, instead forming a band of gray matter underneath the cortical surface (neuronal under-migration) (Guerrini and Dobyns, 2014; Guerrini and Marini, 2006). The echinoderm microtubule-associated protein-like 1 (*EML1*) gene has been associated with subcortical band heterotopia (Kielar et al., 2014; Zaidi et al., 2024). Abnormal mitotic spindle orientation has been observed in *Eml1*-null progenitors, and the ensuing delamination of apical progenitors has been proposed as leading to the ectopias (Bizzotto et al., 2017; Kielar et al., 2014). Although EML1 is a member of a family of proteins that includes six members in both mice and humans (Fry et al., 2016), EML1 is thus far the only member that has been involved in cortical malformations.

COB (formerly referred to as lissencephaly type 2) is characterized by over-migrating neuroblasts passing through breaches in the PBM into the subarachnoid space (Barkovich et al., 2015; Barkovich et al., 2012; Guerrini and Dobyns, 2014; Leventer et al., 2008). There are two alternative pathogenic mechanisms that can result in COB. The most common mechanism involves a weak or disrupted PBM that fails to provide a physical boundary or chemical stop cues to migrating neurons (Radmanesh et al., 2013; van Reeuwijk et al., 2005b). In the alternative mechanism, migrating neurons fail to detect or interpret stop cues and ultimately tear through an intact and functional PBM (Moers et al., 2008). COB is associated with mutations in the *DAG1* gene, encoding for the alpha-dystroglycan protein (Frost et al., 2010; Geis et al., 2013; Riemersma et al., 2015), and those involved in its glycosylation, that is glycosyltransferases *POMT1*, *POMT2*, *FKTN*, *FKRP*, *POMGNT1*, and *LARGE* (Beltrán-Valero de Bernabé et al., 2002; Godfrey et al., 2007; Mercuri et al., 2009; van Reeuwijk et al., 2005a; Willer et al., 2014). These mutations disrupt radial glial basal endfoot binding to the ECM, destabilizing the PBM (Moore et al., 2002). Mutations in ECM components like laminin gamma-1 can also contribute to PBM instability (Halfter et al., 2002). Since the alpha-dystroglycan and laminin gamma-1 proteins are also present in basement membranes other than the PBM, COB is typically observed as one of the phenotypes of a syndrome (Godfrey et al., 2007; Mercuri et al., 2009; Willem et al., 2002). However, COB can also present as an isolated condition, with about 30% of COB cases exhibiting atypical non-syndromic forms with no identified mutations, underscoring the disorder’s significant genetic and clinical heterogeneity (Devisme et al., 2012; Radmanesh et al., 2013).

The EML proteins contain a tandem atypical propeller in EMLs (TAPE) domain containing WD40 repeats that fold into beta-propellers (Richards et al., 2014). The C-terminal TAPE domain in EML1 and EML2 has been shown to bind soluble tubulins (Richards et al., 2014). The N-terminus of EML proteins harbors a coiled-coil, which has been demonstrated in EML2 and EML4 to mediate protein trimerization (Richards et al., 2015). Additionally, in EML1 and EML4, the N-terminal region was shown to be required for association with polymerized microtubules (Pollmann et al., 2006; Richards et al., 2015). Although EML proteins have been proposed to modulate microtubule stability (Eichenmuller et al., 2002; Fry et al., 2016; Houtman et al., 2007; Pollmann et al., 2006), further research is needed to understand the precise functions and contributions of each member of the EML protein family in cellular processes and specifically in neuronal development.

Previous work has shown that when *EML3* is knocked down in cultured cells, chromosomes are misaligned during metaphase, and cells are delayed in the mitotic phase of the cell cycle (Tegha-Dunghu et al., 2008). It was later shown that EML3 is required for recruitment of the augmin protein and gamma-tubulin ring complexes to existing microtubules for microtubule-based microtubule nucleation, a process that enables mitotic spindles to capture metaphase chromosome kinetochores in a timely manner (Luo et al., 2019). These studies define EML3 as a modulator of spindle assembly during cell division. Given the role for EML3 in mitotic spindle formation in cultured cells and given the role for EML1 in controlling spindle orientation in neural progenitors, we hypothesized that the *Eml3* gene may also be required for cortical development.

To examine the possibility that *Eml3* is required during development, we generated *Eml3* null mice. We report that *Eml3* null embryos are developmentally delayed, die perinatally, and have focal neuronal ectopias (FNEs) with neuroblasts that migrate into the overlaying subarachnoid space through a defective PBM. *Eml3* null mice are a model for COB malformation with a defect in ECM remodeling.

## Results

### Generation of *Eml3* null mice

**Figure 1.**
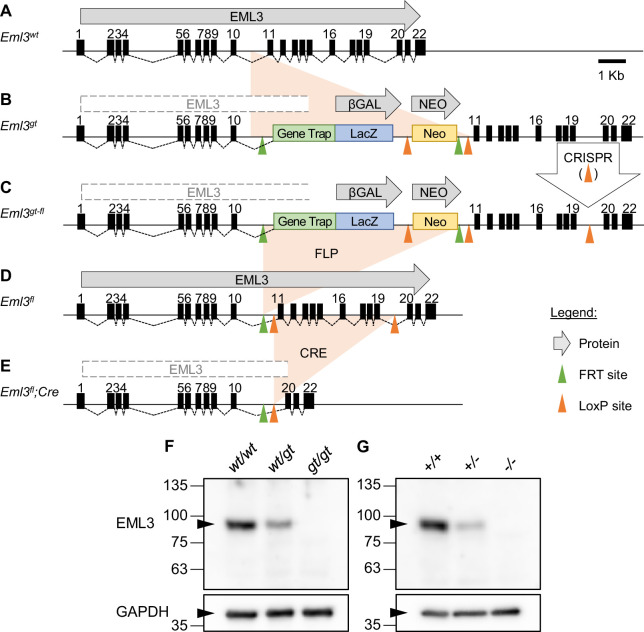
Constitutive and tissue-specific targeting of *Eml3*. (**A**) *Eml3* has 22 coding exons in mouse chromosome 19. (**B**) Constitutive *Eml3* null mice (*Eml3tm1e(EUCOMM)Wtsi* or *Eml3gt*) with a gene trap allele from a EUCOMM ES cell line. (**C**) The gene trap-floxed allele (*Eml3em1Mci* or *Eml3gt-fl*) with a LoxP site inserted into intron 19 (floxed exons 11–19). (**D**) The floxed allele (*Eml3em1.1*Mci or *Eml3fl*) with the gene trap cassette excised. (**E**) The tissue-specific *Eml3* null alleles, (*Eml3fl;Cre*) in the presence of *Cre* recombinases under the control of tissue-specific promoters. The use of a germline-expressed *Cre* driver (*E2a-Cre*) yielded a global *Eml3* null allele named *Eml3em1.2Mci*. Immunoblotting of E15.5 whole embryo lysates with EML3 antibody (EML3-C884) detected no EML3 protein (~90 kDa) in homozygotes for either (**F**) the gene trap allele (*Eml3tm1e(EUCOMM)Wtsi* or *Eml3gt*), or (**G**) the exon 11–19 deletion allele (*Eml3em1.2Mci* or *Eml3*^−^). Heterozygous embryos yielded approximately half the amount of protein as wild-type controls in both mouse lines. GAPDH immunoblotting was used as the protein loading control. Figure 1—source data 1.PDF file containing original TIF images used to prepare Figure 1, panels F and G, indicating the relevant bands and genotypes. Figure 1—source data 2.TIF files of images obtained from Bio-Rad ChemiDoc for immunoblotting displayed in Figure 1F, G.

To characterize the developmental processes requiring the EML3 protein, the mouse *Eml3* gene was targeted. As described in Materials and Methods, the following mice were generated: *Eml3^tm1e(EUCOMM)Wtsi^* or *Eml3*^gt^ (gene trap allele) and *Eml3^em1.2Mci^* or *Eml3*^−^ (knockout allele with exons 11–19 deleted) (Figure 1). Both mouse lines were verified to represent *Eml3* null alleles. The reduction of EML3 protein levels in heterozygotes (*Eml3^wt/gt^* and *Eml3^+/−^*) and its elimination from homozygous embryos (*Eml3^gt/gt^* and *Eml3^−/−^*) was confirmed by immunoblotting (Figure 1F, G). Mice were backcrossed to C57BL/6J background for at least two generations prior to conducting experiments. Experimental mice were produced by intercrossing heterozygotes. All experiments were performed using both male and female littermates, and all data shown are pooled from both sexes after verification that there were no sex-dependent differences.

Three main phenotypes were observed in *Eml3* null mice: embryonic development delay, brain defect, and perinatal lethality (Table 1). All phenotypes are recessive, heterozygous mice (*Eml3^+/−^*) being indistinguishable from their *Eml3^+/+^* littermates.

**Table 1.** Phenotypes observed in *Eml3* null mice.

Phenotype	Sub-phenotype	Age when observed^*^	Penetrance of phenotype vs *Eml3* genotype		
			*Eml3^+/+^*	*Eml3^+/−^*	*Eml3^−/−^*
**Embryo development delay**	Small embryo and placenta size^†^	E8.5–E18.5	2/41 (4.9%)	1/75 (1.3%)	23/23 (100%)
	Fewer somite pairs^‡^	E8.5–E12.5	3/25 (12%)	4/51 (8%)	11/20 (55%)
	Delayed neurogenesis^§^	E10.5–E11.5	0/10 (0%)	0/1 (0%)	8/11 (72.7%)
	Thin corpus callosum^¶^	E18.5	0/8 (0%)	0/1 (0%)	10/10 (100%)
**Brain defect**	Focal neuronal ectopias	E11.5–E18.5	0/10 (0%)	n.d.	10/14 (71.4%)
**Perinatal lethality**	Failure to inflate lungs^**^	E18.5	5/24 (20.8%)	5/33 (15.2%)	6/10 (60%)
	Survival up to weaning age^††^	PN18–24	n.d.	355 Het vs 191 WT (~7% Het lethality)	11 KO vs 191 WT (~94% KO lethality)

* Embryonic (E) or postnatal (PN) ages at which the phenotype was observed. When a range of ages was studied, the penetrance data shown in the table is from the underlined age.

† Embryo sizes were measured as area of the section corresponding to the sagittally bisected E8.5 and E10.5 embryos or as weights for E11.5 and E18.5 embryos. E18.5 weight analyses shown, with 15% lighter weight vs average of heterozygote littermates as the threshold for small size. 100% penetrance for low weight of *Eml3* null embryos was observed at E12.5–E18.5. *Eml3* null placentas are small at E10.5–E18.5.

‡ Somite pairs were counted under a dissection microscope for E8.5–E12.5 embryos. E12.5 data shown, with a minimum difference of 1.5 somite pairs vs the average for heterozygous littermates as the threshold for delay.

§ Delayed neurogenesis was determined by staining E10.5–E11.5 frontal cortices with markers for neuroepithelial progenitors (PAX6 and SOX1), intermediate neuronal progenitors (TBR2), and postmitotic projection neurons (TBR1). E11.5 data shown as the number of embryos with at least a 20% lower percentage of TBR2-positive cells in their frontal cortex as compared to a control littermate.

¶ Corpus callosum (CC) thicknesses were measured in Nissl-stained coronal sections of E18.5 whole brains. Pairwise comparisons among littermates determined that all *Eml3* null embryos had a disproportionally thinner CC than their littermates even when normalizing for the overall smaller size of the embryos.

** Air breathing and lung floating assays were performed on E18.5 embryos collected by cesarean section. The pups were observed and filmed for 90 min. At the end of the observation period, all mice were euthanized, and the lungs were removed and placed in PBS for the lung floating assay.

†† We estimate the number of missing mice at weaning age by comparing the number of *Eml3* null and heterozygous mice to the number of weaned wild-type mice. Given that we analyze litters from heterozygote intercrosses and that we expect a ratio of 1 WT:2Het:1KO, and that we obtained a total of 191 WT mice, we were expected to recover 382 Het and 191 KO mice.

### Perinatal lethality in *Eml3* null mice

To determine the phenotype of *Eml3* null mice, we intercrossed heterozygotes (*Eml3*^+/−^) and characterized the offspring that survived until weaning age (approximately 21 days old). Out of 557 weaned mice, only 11 *Eml3*^−/−^ pups were obtained (Tables 1 and 2). *Eml3*^−/−^ mice, therefore, represent 2% of all weaned mice, in contrast with the expected 25% if viable. Notably, most of the rare *Eml3* null survivors were runted, with 32% average lower body weight than littermate controls, and 25% had hydrocephalus. The lethality phenotype is recessive since heterozygotes (*Eml3*^+/−^) are found approximately in the expected proportion of 2:1 vs wild-type mice (*Eml3*^+/+^). We conclude that a majority of *Eml3* null mice die before weaning age, and the phenotype is recessive.

To define the timepoint of lethality, timed heterozygote intercross matings were set up and genotype ratios were determined at several stages of embryonic development (Table 2). Mendelian ratios were obtained at all stages studied according to chi-square analyses. We conclude that *Eml3* null mice die mostly during or after birth. Dead *Eml3* null pups were observed within 48 hr following birth. To gain insight into the underlying cause of perinatal death, we closely monitored the fate of litters obtained via C-section at E18.5. The observation period lasted for 1.5 hr after surgical delivery. 6/10 (60%) *Eml3* null neonates and 10/57 (18%) control littermates showed severe respiratory distress and died within 30–40 min after C-section (Table 1). These neonates had strong respiratory reflexes but remained cyanotic and died of acute respiratory failure. Immediately following the observation period, the lungs were dissected from the neonates and immersed in saline. Lungs from neonates that had not survived sank in the saline since they had not inflated with air. Failure to inflate lungs can be a consequence of lung immaturity and is encountered in several knockout mouse models (Turgeon and Meloche, 2009). Indeed, histological analysis of serial sections of E18.5 *Eml3* null embryos revealed immaturity of the lungs in a significant portion of the *Eml3* null embryos examined (3/4 *Eml3* null embryos had not reached saccular stage in lung development, a proportion higher than what was observed in control mice). No skeletal defect, nor structural defect in the diaphragm and intercostal muscles were observed in the thorax of *Eml3* null embryos. The analyses did not reveal any gross morphological anomaly in other internal organs. Overall, the neonate characterization data suggests that the postnatal lethality of *Eml3* null mice was at least in part due to respiratory distress due to lung immaturity.

**Table 2.** Lethality in *Eml3* null mice.

Mouse age	No. of animals	*Eml3* genotype			p value^*^
		+/+	+/−	−/−	
Embryos (dpc)^†^					
11.5	339	82	167	90	0.7980
12.5	113	26	61	26	0.6988
13.5	151	47	67	37	0.1981
18.5	144	43	77	24^‡^	0.0576
					
Weaned mice^§^	557	191	355	11	<0.0001
					

* A *χ*^2^ goodness of fit test was performed at each stage for genotype ratios obtained from heterozygote intercrosses.

† Days post coitum.

‡ Some advanced resorptions observed but not collected.

§ Mice were weaned at about three weeks of age.

### Global developmental delay in *Eml3* null embryos

**Figure 2.**
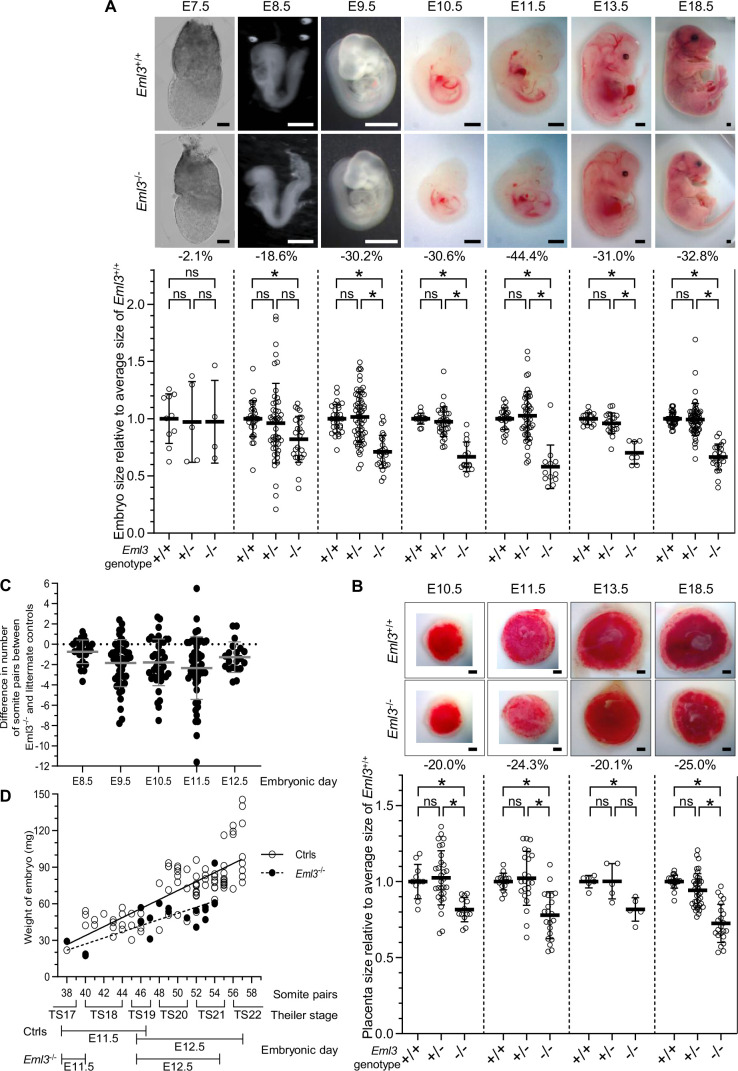
*Eml3*^−/−^ embryos are smaller than control littermates and are developmentally delayed. At each gestational age between E8.5 and E18.5, the *Eml3^−^*^/−^ embryos (**A**) and their placentas (**B**) (E10.5–E18.5) are significantly smaller than their *Eml3*^+/+^ and *Eml3*^+/−^ control littermates. The size was measured either by area (E7.5–E10.5 embryos; E10.5–E18.5 placentas) or weight (E11.5–E18.5 embryos); for each litter analyzed, the size of each embryo or placenta was measured relative to the average of the control embryos (*Eml3*^+/+^ and *Eml3*^+/−^) and then normalized to the average of the *Eml3*^+/+^ embryos. The average percent size difference is shown below each image (*n* = 5–32). Dunn’s pairwise comparison, *p < 0.05, ns = not significant. Scale bars, 100 µm for E7.5, 500 µm for E8.5, and 1 mm for E9.5–E18.5. (**C**) At each gestational age between E8.5 and E12.5, the *Eml3^−/−^* embryos have significantly fewer somite pairs than *Eml3*^+/+^ and *Eml3*^+/−^ control littermates; average difference in number of somite pairs ± SD shown (*n* = 20–50). (**D**) Weight of embryos was plotted against the number of somite pairs counted for *Eml3^−/−^* and control embryos. Linear regression was used to fit the datapoints; *Eml3*^−/−^ embryos are significantly smaller (p < 0.0001) than control embryos with the same number of somite pairs.

While characterizing embryos and neonates, we noted that *Eml3* null mice were smaller than control littermates (Figure 2A and data not shown). Except for their size, *Eml3* null embryos were morphologically normal throughout embryonic development (Figure 2A). To determine if the lung immaturity and small size of *Eml3* null neonates are an indication of global developmental delay, we characterized *Eml3* null embryos at different stages of development. Timed matings were established between *Eml3* heterozygotes and embryos were examined at specific ages, measured in days post coitum. At E7.5 no significant size difference was observed between *Eml3* null and control embryos (Figure 2A). However, on embryonic days E8.5 to E18.5, they were 19–44% smaller in size (Figure 2A). The placentas were also examined from E10.5 to E18.5 and found to be 20–25% smaller in size (Figure 2B). In addition, delayed appearance of intermediate progenitors (Figure 4A) and neuroblasts, delayed saccular stage of lungs at E18.5, and a thinner corpus callosum at E18.5 (Table 1) suggest that *Eml3* null embryos are delayed in development. Indeed, within a single litter, *Eml3* null embryos had fewer somite pairs when compared to control littermates at all stages analyzed (E8.5–E12.5; Figure 2C). At E8.5, shortly after the onset of somitogenesis, there was on average one somite pair fewer in *Eml3* null embryos compared to control littermates (Figure 2C). At E9.5, E10.5, and E11.5, a constant two somite pair mean reduction in the total number of somite pairs was observed. Interestingly, comparing the weight of *Eml3* null embryos to control embryos with the same number of somite pairs, that is, stage-matched, revealed that *Eml3* null embryos were still smaller than control embryos (Figure 2D). This suggests that, in addition to the developmental delay, the *Eml3* null embryos are growth restricted. We conclude that *Eml3* null mice are developmentally delayed starting at E8.5 and are smaller than stage-matched control littermates (growth restricted).

### FNEs in *Eml3* null embryos

#### Regional lamination defects in Eml3 null mouse brains

**Figure 3.**
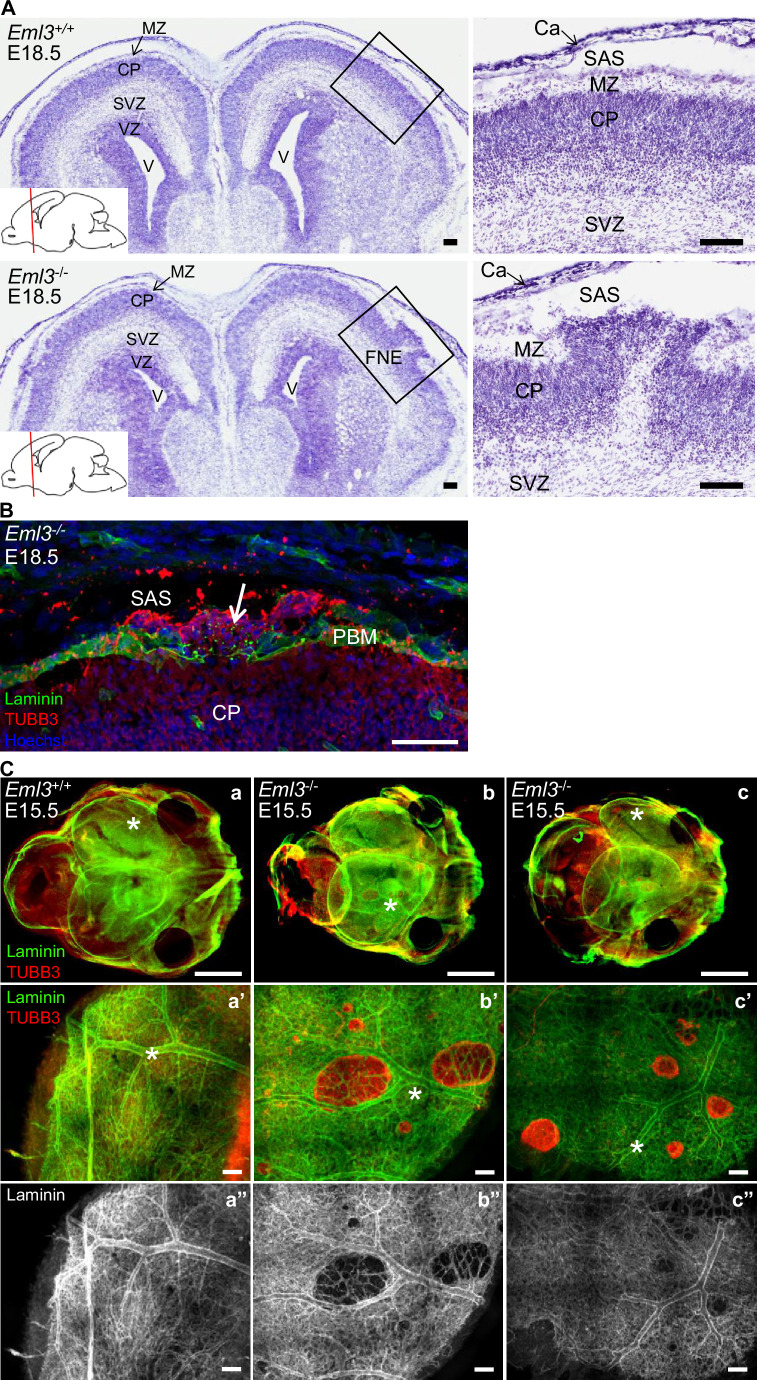
Focal neuronal ectopias (FNEs) in *Eml3^−/−^* embryos. (**A**) Nissl (cresyl-violet) staining of coronal sections of forebrains from E18.5 *Eml3^+/+^* and *Eml3^−/−^* embryos. The *Eml3^+/+^* sections in the top panels show normal brain architecture with a close-up view in the panel on the right. An FNE is present in the section of the *Eml3^−/−^* cortex shown in the bottom panels. The close-up in the bottom-right panel shows over-migrating neurons (dark cresyl-violet staining) extending past the marginal zone and into the subarachnoid space. Ca, calvarium; CP, cortical plate; MZ, marginal zone; SAS, subarachnoid space; SVZ, subventricular zone; V, ventricle; VZ, ventricular zone. Scale bars, 100 µm. (**B**) Immunolabeling of an E18.5 *Eml3^−/−^* mouse brain section for laminin (green), a major component of the extracellular matrix of the pial basement membrane, and for neuron-specific tubulin beta-3 (TUBB3, red). The pial basement membrane is disrupted at the FNEs, with neurons (TUBB3+) over-migrating into the subarachnoid space. Laminin speckles are observed (arrow). CP, cortical plate; PBM, pial basement membrane; SAS, subarachnoid space. Scale bar, 50 µm. (**C**) Heads of E15.5 *Eml*3^+/+^ and *Eml3^−/−^* embryos were immuno-stained and cleared before imaging with a fluorescence stereomicroscope (**a–c**) or with a confocal microscope (**a’–c”**). Immunostaining was performed with antibodies against the ECM protein laminin (green in **a–c** and **a’–c’**, white in **a”–c”**) and a marker of terminally differentiated neurons, tubulin beta-3 (TUBB3, red). The asterisks point to the same area in the high magnification confocal microscope images as in the low magnification stereomicroscope images. Scale bars, 1 mm (**a–c**) and 0.1 mm (**a’–c”**).

To determine if the *Eml3* null brains have gross morphological defects, we stained coronal and sagittal brain sections for the Nissl substance (cresyl violet stain) that highlights neuronal structure. In E18.5 *Eml3* null mouse embryos, we observed ectopic clusters of cells within the marginal zone and the subarachnoid space of the dorsal telencephalon (Figure 3A). These ectopias were observed in 10/14 *Eml3* null brains examined (71.4% penetrance) and absent from all 10 control *Eml3^+/+^* mice analyzed (Table 1). The cells in the ectopic clusters were immunoreactive when stained with antibodies to the pan-neuronal marker tubulin beta-3 (TUBB3; Figure 3B), identifying the cells as neurons and establishing the ectopic clusters as FNEs. Laminin immunostaining, a major component of the PBM, indicated that the PBM was fragmented at the FNEs (laminin speckles; Figure 3B). Below each FNE, the cortical architecture was disorganized, suggesting that a cohort of neurons had over-migrated and traversed the PBM (Figure 3A).

To analyze the spatial distribution of FNEs in developing brains, immunostained heads were cleared and imaged (Figure 3C). Whole E15.5 heads were immunostained with laminin to highlight the PBM and tubulin beta-3 to highlight post-mitotic neuroblasts. In addition, *Eml3* null E18.5 brain serial sections were stained with cresyl violet (Figure 3A). The number of FNEs in *Eml3* null brains varied from none to 12; the FNEs varied in size and were randomly distributed rostrocaudally in both forebrain hemispheres (Figure 3A, C). FNEs were restricted to the dorsal aspect of the telencephalon, whereas medial cortical fields near the interhemispheric fissure and ventral areas were unaffected. No histological abnormalities were found in other brain regions.

#### Normal neurogenesis in Eml3 null dorsal telencephalon

**Figure 4.**
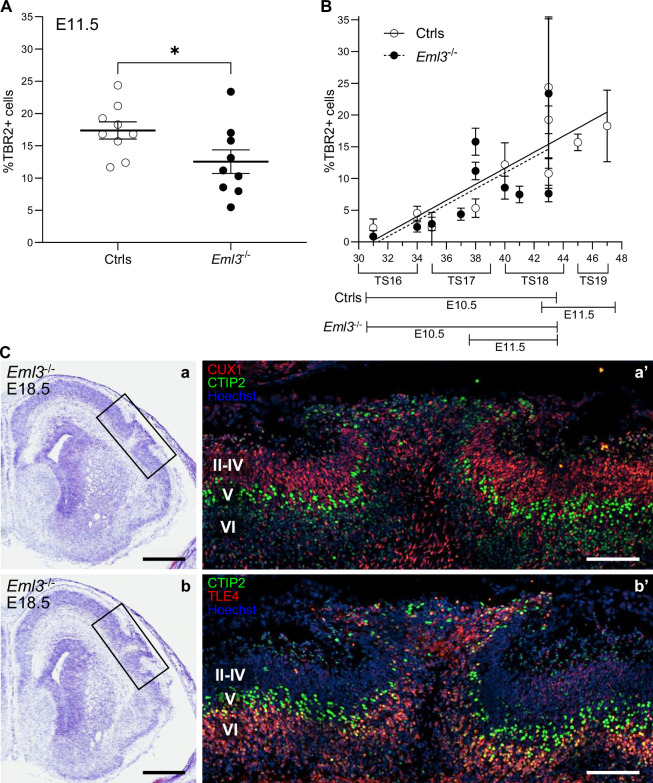
*Eml3^−/−^* embryos are developmentally delayed vs littermate controls, but the onset and progression of neurogenesis are equal in somite-matched *Eml3^−/−^* and control forebrains; additionally, neurons of all cortical plate layers are present in *Eml3^−/−^* focal neuronal ectopias (FNEs). (**A**) The onset and progression of neurogenesis are delayed in *Eml3*^−/−^ vs littermate control forebrains. Forebrains from embryos collected at E11.5 were cryosectioned and processed for indirect immunofluorescence with markers for cell populations whose relative abundances indicate the onset and progression of neurogenesis. The percentage of TBR2-positive intermediate progenitors was determined. *Eml3^+/+^* and *Eml3^+/^*^−^ data are pooled as controls (Ctrls) vs *Eml3^−/−^* embryos. The total number of cells in the embryonic brain section was determined with the nuclear stain Hoechst. Each dot on the graph represents the mean percentage of cells positive for TBR2 from analysis of 2–4 sections per embryo. Error bars represent SEM. Pairwise comparison using Student’s *t*-test, *p < 0.05. (**B**) The onset and progression of neurogenesis are equal in somite-matched *Eml3^−/−^* and control forebrains. Forebrains from embryos collected at E10.5 and E11.5 were cryosectioned and processed for indirect immunofluorescence with markers for cell populations whose relative abundances indicate the onset and progression of neurogenesis. The percentage of TBR2-positive intermediate progenitors was determined and is plotted against the number of somite pairs counted in each embryo. The total number of cells in the embryonic brain section was determined with the nuclear stain Hoechst. Each dot on the graph represents the mean percentage of cells positive for TBR2 in an individual embryo. Error bars represent SD from analysis of 2–4 sections per embryo. *Eml3^+/+^* and *Eml3^+/^*^−^ data are pooled as controls (Ctrls) vs *Eml3^−/−^* embryos. Shown below the graphs is the Theiler stage of development that corresponds to the number of somite pairs counted. Also indicated is the age of the embryos in days post-coitum. (**C**) Neurons of all cortical plate layers are present in *Eml3^−/^*^−^ FNEs at E18.5. (**a, b**) Nissl (cresyl-violet) staining of serial coronal sections through a single FNE. (**a’, b’**) Immunolabeling of adjacent sections of the same FNE with markers of cortical plate layers. (**a’**) CUX1 immunostaining of late-born upper layer neurons (II–IV) and CTIP2 immunostaining of intermediate layer neurons (**V**). (**b’**) TLE4 immunostaining of early-born deeper layer neurons (VI) and CTIP2 immunostaining. Scale bars, 500 µm (**a, b**) and 100 µm (**a’, b’**).

To establish whether the FNEs observed in *Eml3* null mice are a consequence of disturbed neurogenesis, as determined for the subcortical band heterotopias present in *Eml1* null mice (Bizzotto et al., 2017; Kielar et al., 2014), we immunostained *Eml3* null and control forebrains with key markers of neurogenesis. E10.5 and E11.5 embryo cryosections were immunostained for TBR2 and TBR1, which are markers of intermediate progenitors and of post-mitotic neural precursors, respectively. The appearance of both cell types defines the onset of neurogenesis. We calculated the percentage of TBR2 and TBR1 immunoreactive cells present in each forebrain cryosection at specific embryonic ages. The appearance of intermediate progenitors was delayed in *Eml3* null mice (TBR2 at E11.5, Figure 4A). Analyses at additional embryonic ages and with TBR1 staining indicated that neurogenesis milestones were reached at a later age in *Eml3* null embryos (data not shown). Since *Eml3* null mice are developmentally delayed as determined by the number of somites at a specific age (Figure 2C), we then plotted the average percentage of TBR2+ cells against the number of somite pairs for each embryo, allowing for a comparison of stage-matched *Eml3* null and control embryos. TBR2+ cells appeared at the same stage, and their prevalence increased at the same rate in *Eml3* null and control embryos (Figure 4B and data not shown for TBR1). We tested two additional markers, SOX1 and PAX6, that are expressed sequentially during early neurogenesis. The transition from *Sox1* to *Pax6* expression in mice marks the progression from early neuroepithelium to more differentiated radial glial cells during early embryonic development, typically occurring around embryonic day E8.5–9.5. *Sox1* is expressed first, marking the neural plate*. Pax6* is expressed later, driving the transition to radial glia and neuronal differentiation (Suter et al., 2009). No differences in SOX1 and PAX6 staining were observed between stage-matched *Eml3* null and control embryos (data not shown). Thus, *Eml3* null mice have reached the same neurogenesis milestones as their WT counterparts when they have the same number of somites (Figure 4B). Given that *Eml1* null radial glia have perturbed mitotic spindle angles, we measured the angle of mitotic spindles in *Eml3* null and control apical progenitors and saw no differences when the embryos were stage-matched (data not shown). We conclude that the onset and progression of neurogenesis are normal in *Eml3* null mice.

#### Ectopic neurons comprise neurons from all cortical layers

Having determined that the cortical defect in *Eml3* null mice is not associated with abnormal neurogenesis, we aimed to identify what other pathological mechanism is responsible for the *Eml3* null FNEs. Our first aim was to establish the onset of neuronal over-migration. Since neurons born at different developmental stages express different markers (Greig et al., 2013; Noctor et al., 2004), identifying the neurons present within the FNEs gives an estimate of the onset of neuronal over-migration. To determine the cellular composition of the ectopias observed at E18.5, we performed immunostaining with layer-specific markers: CUX1 for layers II–IV (Nieto et al., 2004), CTIP2 for layer V (Arlotta et al., 2005), and TLE4 for layer VI (Yao et al., 1998). Neurons immunoreactive for each of the three neuronal markers CUX1, CTIP2, and TLE4 were detected in the ectopias (Figure 4C). Therefore, the ectopic cells in the *Eml3* null FNEs are neurons from deep and superficial cortical layers. These observations suggested that the developmental defect that leads to the FNEs in *Eml3* null mice is present when the first radially migrating neuroblasts of the cortical plate reach the PBM.

In addition, analysis of neuronal marker layering determined that cortical lamination outside of the FNEs is normal (Figure 4C), indicating that neurogenesis and neuroblast radial migration are normal outside of the FNEs.

#### Neuroblasts from the first radially migrating cohort over-migrate into the subarachnoid space

**Figure 5.**
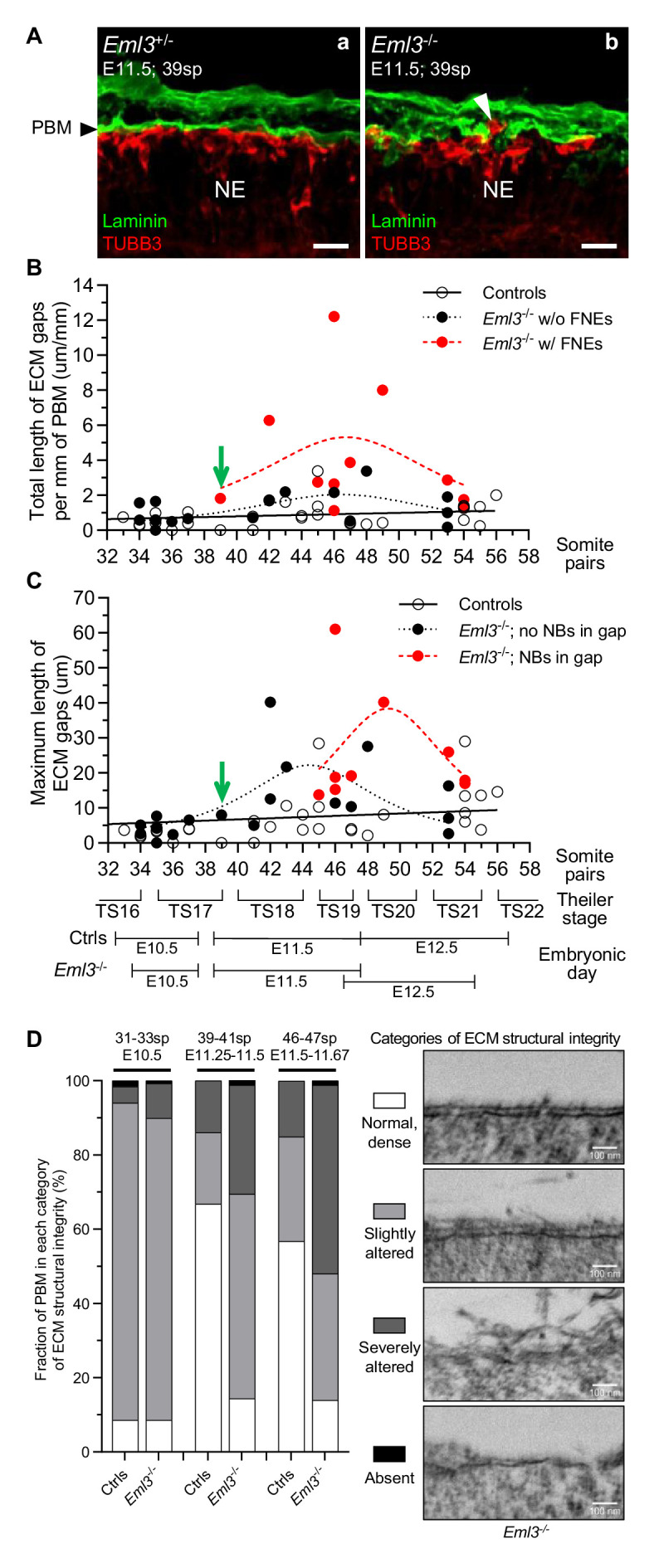
Onset of neuronal over-migration and structural integrity of pial basement membrane (PBM) ECM. (**A**) Double immunostaining of laminin (green) and tubulin beta-3 (TUBB3, red) at 39 somite pair (sp) embryonic stage (TS17; collected at E11.5). Scale bars, 20 µm. (**a**) In a heterozygous brain, the PBM is continuous and migrating neuroblasts are observed underneath, whereas in an *Eml3*^−/−^ embryo (**b**) a gap in the ECM and ectopic neuroblasts (arrowhead) were detected. (**B, C**) Coronal head sections of control and *Eml3*^−/−^ embryos at different developmental stages were stained for laminin and tubulin beta-3. The length of gaps in the ECM was measured and the presence of over-migrating neuroblasts (NBs) was noted. The total length of PBM evaluated was measured for normalization. The green arrow points to the *Eml3*^−/−^ embryo that had a focal neuronal ectopia (FNE) at the earliest developmental stage, that is, 39 somite pairs. (**B**) For each embryo, the total length of gaps per mm of PBM analyzed was plotted against the number of somite pairs counted. The *Eml3*^−/−^ embryos with and without FNEs were plotted separately. (**C**) For each embryo, the length of the largest gap observed was plotted against the number of somite pairs counted. For the *Eml3*^−/−^ embryos, the largest gaps with and without over-migrating NBs through the gap were plotted separately. No over-migrating NBs were ever observed in gaps of control embryos. (**D**) Transmission electron microscopy was performed on sections of *Eml3^−/^*^−^ and control embryos (Ctrls; *Eml3^+/+^* and *Eml3^+/^*^−^) matched by developmental stage. Three stages were analyzed (31–33, 39–41, and 46–47 somite pairs; sp). The embryo ages in days post-coitum are indicated. The total length of PBM was measured in each TEM micrograph. The ECM length was then subdivided into regions with different structural integrity. Normal, dense: sharp, straight, electron-dense ECM. Slightly altered: thicker, diffuse, less dense ECM. Severely altered: delaminating ECM, with electron-dense material shedding off a diffuse PBM. Absent: regions with no ECM overlaying the NEP basal end-feet. Representative micrographs shown. At the 31–33 sp stage, control ECM was 8.6 ± 7.8% normal (dense), 85.4 ± 8.0% slightly altered, 4.3 ± 3.6% severely altered, and 1.6 ± 1.4% absent, whereas *Eml3^−/^*^−^ ECM was 8.5 ± 7.0% normal (dense), 81.4 ± 2.9% slightly altered, 9.3 ± 8.1% severely altered, and 0.7 ± 1.1% absent. At the 39–41 sp stage, control ECM was 66.8 ± 6.3% normal, 19.3 ± 9.0% slightly altered, and 13.9 ± 15.2% severely altered, whereas *Eml3^−/^*^−^ ECM was 14.4 ± 6.7% normal, 55.1 ± 23.8% slightly altered, 29.3 ± 30.2% severely altered, and 1.2 ± 0.4% absent. At the 46–47 sp stage, control ECM was 56.8 ± 9.1% normal, 28.2 ± 4.0% slightly altered, and 15.0 ± 5.1% severely altered, whereas *Eml3^−/^*^−^ ECM was 13.9 ± 5.9% normal, 34.1 ± 7.2% slightly altered, 50.8 ± 11.6% severely altered, and 1.2 ± 1.6% absent. Scale bars, 100 nm.

To identify the developmental stage at which the first neuronal over-migration events occur, we looked at high spatial and temporal resolution for gaps in the PBM and the presence of over-migrating neurons. Our analysis revealed that the earliest occurrence of a gap in the PBM with neuronal over-migration was in an *Eml3* null embryo with 39 somite pairs (TS17, collected at E11.5; Figure 5A). Notably, the few tubulin beta-3 immunoreactive neuroblasts observed immediately below the PBM at the 39-somite pair stage represent the first cohort of cells to reach the PBM through radial migration.

**Figure 5—figure supplement 1.**
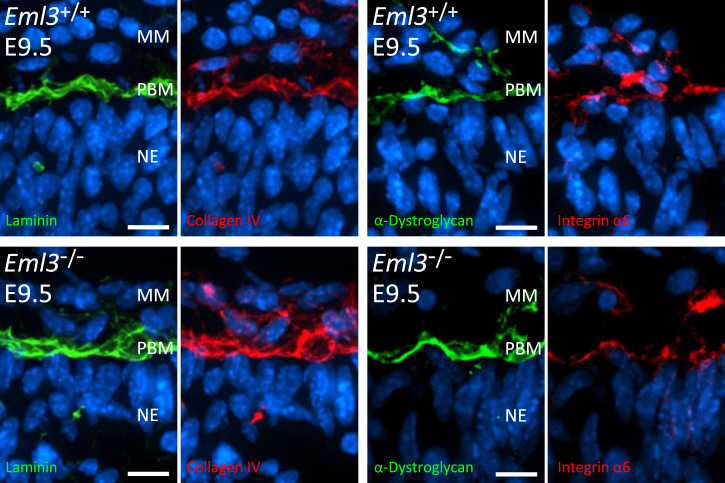
Components of the PBM in stage-matched E9.5 *Eml3*^+/+^ and *Eml3*^−/−^ embryos. Immunolabeling of the pial basement membrane on sections of developing brains from stage-matched E9.5 *Eml3*^+/+^ (top) and *Eml3*^−/−^ (bottom) mouse embryos. Antibodies against the major components of the extracellular matrix of the PBM: laminin (green, left) and collagen IV (red, left), as well as for the major ECM component receptors expressed on the end-feet of neuroepithelial cells: α-dystroglycan (green, right) and integrin α6 (red, right) were used. MM, meningeal mesenchyme; NE, neuroepithelium; PBM, pial basement membrane. Scale bar, 10 µm.

#### Increased PBM discontinuity in Eml3 null embryos coincides with the arrival of radially migrating neuroblasts

Over-migration of neuroblasts into the subarachnoid space can occur because of defects in the integrity of the PBM, which can no longer halt the migrating neurons (Radmanesh et al., 2013; van Reeuwijk et al., 2005a). Alternatively, neurons unable to detect or integrate migration stop cues can punch through a normal PBM (Moers et al., 2008). The presence of defects in PBM structure prior to the arrival of radially migrating neuroblasts can distinguish between the two possible mechanisms for FNE formation.

As a first step in the characterization of the *Eml3* null PBM, we immunostained for laminin, collagen IV, α-dystroglycan, and integrin α6. No major differences were observed (Figure 5—figure supplement 1).

To further characterize the PBM of *Eml3* null embryos, we quantified gaps in the laminin immunoreactive PBM in embryos at developmental stages between 33 and 56 somite pairs (Figure 5B, C). The cross-sectional length of each gap was measured. The total length of all the gaps observed in the PBM of an embryo (μm per mm of PBM analyzed, a measure of gap burden) was calculated and plotted against the number of somite pairs counted for that embryo (Figure 5B). The cross-sectional length of the largest gap per embryo (maximum cross-sectional length in μm) was also plotted against the number of somite pairs (Figure 5C). In control brains, PBM gaps were rare (average gap burden of 1.1 μm per mm of PBM; Figure 5B) and small (average maximum gap size below 10 μm; Figure 5C) at all developmental stages examined. The total and maximum size of gaps did not significantly differ between control and *Eml3* null embryos of fewer than 39 somite pairs (Figure 5B, C). However, in embryos with more than 39 somite pairs, the total length of gaps per mm of PBM was significantly larger in *Eml3* null embryos than in control embryos (Figure 5B). The gap burden in μm per mm PBM peaked at the 47-somite pair stage in *Eml3* null embryos and was larger in *Eml3* null embryos with FNEs (about 5.3 μm gap per mm of PBM) than in *Eml3* null embryos without FNEs (2.0 μm gap per mm of PBM). When maximum gap size was analyzed, *Eml3* null embryos had significantly larger gaps than control embryos after the 39-somite pair stage (Figure 5C). In *Eml3* null embryos, some of the largest PBM gaps did not have over-migrating neuroblasts. Indeed, in the *Eml3* null embryo with the earliest observed FNE, the over-migrating neuroblasts were not within the largest gap found in that embryo. Best-fit curves for maximum ECM gap size in *Eml3* null embryos peaked at 22 μm at the 44-somite pair stage when there were no ectopic neuroblasts visible in the gap. However, when there were ectopic neuroblasts within the gap, it reached 38 μm at the 49-somite pair stage (Figure 5C).

Thus, *Eml3* null embryos have larger gaps and a higher gap burden than control embryos. The gap burden is higher in *Eml3* null embryos that have FNEs than in *Eml3* null embryos that do not have FNEs. Also, gaps with over-migrating neuroblasts are larger than gaps that do not have over-migrating neuroblasts. Importantly, PBM gaps without over-migrating neuroblasts are larger in *Eml3* null than in control embryos. Hence, the *Eml3* null PBM is defective in the absence of over-migrating neuroblasts, and the hypothesis in which neuroblasts punch through an intact PBM in *Eml3* null developing cortex can therefore be excluded.

### Structural abnormalities in the ECM of the Eml3 null PBM

Since the differences in gap size and gap burden are not yet significant when the first FNEs appear, we hypothesized that neuroblasts pass through the PBM when the structural defects are not detectable through laminin immunostaining. We therefore looked for subtle structural abnormalities of the PBM before the onset of FNEs. To analyze the structure of the PBM at a higher resolution, transmission electron microscopy (TEM) was performed on sections of control and *Eml3* null embryos. Brains were analyzed at three developmental stages: at 31–33 somite pairs, before the onset of FNEs; at 39–41 somite pairs, the onset of FNEs; and at 46–47 somite pairs, the stage when the gap burden was largest in *Eml3* null embryos. The total length of PBM was measured in each TEM micrograph and subdivided into regions with different structural integrity (Figure 5D). A sharp, straight, electron-dense ECM was categorized as normal and dense. A thicker, diffuse, less dense ECM was categorized as slightly altered. A delaminating ECM, with electron-dense material shedding off a diffuse PBM was categorized as severely altered. Regions with no ECM overlaying the neuroepithelial basal endfeet were categorized as absent. At the 31–33 somite pair stage, the immature PBM ECM is not yet fully condensed; therefore, for both genotypes, most of the ECM was categorized as slightly altered given its low density. Percentages of the PBM that were assigned to each category of structural integrity were significantly different between *Eml3* null and control mice at two of the developmental stages studied. Although no differences in the ECM morphology were observed at the 31–33 somite pair stages, a higher proportion of structurally disrupted ECM in *Eml3* null embryos both at the 39–41 somite pair and at the 46–47 somite pair stages was observed (Figure 5D). Importantly, the morphology and spacing of the basal endfeet of neural progenitors were not different in *Eml3* null and control embryos according to the TEM micrographs at the stages studied. Thus, we conclude that, at the onset of FNEs, the structural integrity of the PBM is compromised, with a structurally abnormal ECM.

### EML3 is expressed in the tissues that form and maintain the PBM

**Figure 6.**
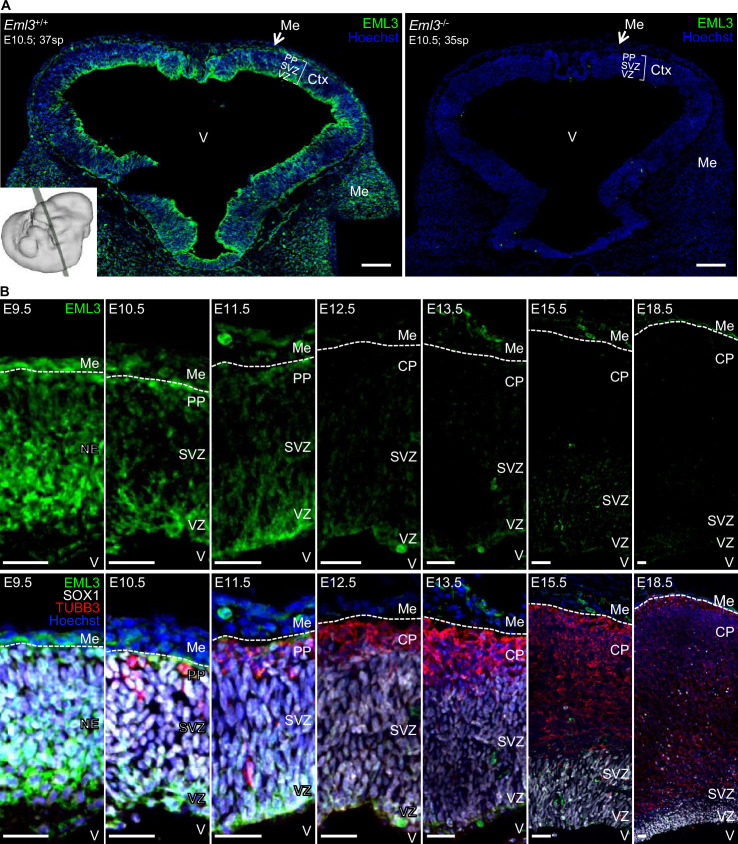
EML3 is expressed in the tissues that form and maintain the pial basement membrane (PBM). (**A**) EML3 protein expression (green) as determined by indirect immunofluorescence on a coronal cryosection of an E10.5 *Eml3*^+/+^ embryo head. This developmental timepoint, 37 somite pairs, immediately precedes the appearance of the first focal neuronal ectopia (FNE) in *Eml3*^−/−^ embryos (at 39 somite pairs). An *Eml3^−/−^* littermate with 35 somite pairs was included as a control for antibody staining specificity. Ctx, cerebral cortex; Me, mesenchyme; PP, preplate; SVZ, subventricular zone; VZ, ventricular zone; V, ventricle. Hoechst nuclear stain (blue). Scale bars, 100 µm. (**B**) EML3 protein expression (green) during forebrain development as determined by indirect immunofluorescence on E9.5–E18.5 *Eml3*^+/+^ embryo cryosections. In the bottom panels, the same EML3-stained section is shown with additional immunostaining for markers of neural progenitor cells (SOX1, white) and of post-mitotic neuronal cells (TUBB3, red), as well as Hoechst nuclear stain (blue). The dotted line indicates the location of the PBM, which separates mesenchyme (Me) and cerebral cortex whose cell composition changes during development. At E9.5, the cortex is spanned by neuroepithelial cells (NE). At E10.5 and E11.5, the neuroepithelial progenitors are differentiating into radial glial cells, and the cortex can now be divided into ventricular zone (VZ), subventricular zone (SVZ), and the post-mitotic neurons of the preplate (PP). At E12.5–E18.5, radially migrating neurons are populating the region below the PBM and forming the developing cortical plate (CP). V, ventricle. Scale bars, 25 µm.

To determine if the EML3 protein is expressed in the cells that secrete and stabilize the ECM of the PBM, EML3 immunostaining was performed on E10.5 embryo heads (Figure 6A). E10.5 embryos with 35–37 somite pairs are at the developmental stage that immediately precedes the onset of PBM defects and FNEs. The antibody specificity was validated with an *Eml3* null littermate. At this stage, the EML3-specific signal is ubiquitous, including the PBM-forming mesenchyme and neuroepithelial cells. Neuroepithelial cells span the entire cerebral cortex at this stage; however, the EML3 signal is strongest near the ventricles, suggesting a preference for an apical subcellular localization in those polarized cells.

To further localize the EML3 protein in the PBM-forming cells during forebrain development, coronal sections of embryonic mouse heads at E9.5–E18.5 were stained with anti-EML3 antibody. EML3 immunoreactivity was detected in the developing brain cortex and mesenchyme at E9.5–E18.5 (Figure 6B), but the levels were highest at the stages that precede the onset of FNEs (E9.5 and E10.5). EML3 expression levels decrease over time, with a marked decrease between E11.5 and E12.5, when neuroepithelial cells are completing differentiation into radial glia (Götz and Huttner, 2005; Noctor et al., 2008). By E18.5, EML3 protein expression was limited to the thin mesenchymal cell layer and the ventricular zone (VZ) of the brain cortex, where SOX1-positive precursors are still present. In both neuroepithelial (E9.5–E11.5) and radial glial cells (E11.5–E18.5), the subcellular localization of the EML3 protein was predominantly apical. In neuroepithelial cells (E9.5–E11.5), the second strongest EML3 signal was in the basal endfeet, which are part of the PBM. At E10.5 and E11.5, co-staining with a marker for post-mitotic neurons, TUBB3, revealed that EML3 protein is also expressed in early migrating neurons, albeit at lower levels than in the neural precursors (Figure 6B). EML3 is therefore expressed in the cells that secrete and stabilize the ECM components of the PBM, and the timing of the EML3 expression overlaps with the onset of the *Eml3* null PBM defects.

### EML3 protein interactions

**Figure 7.**
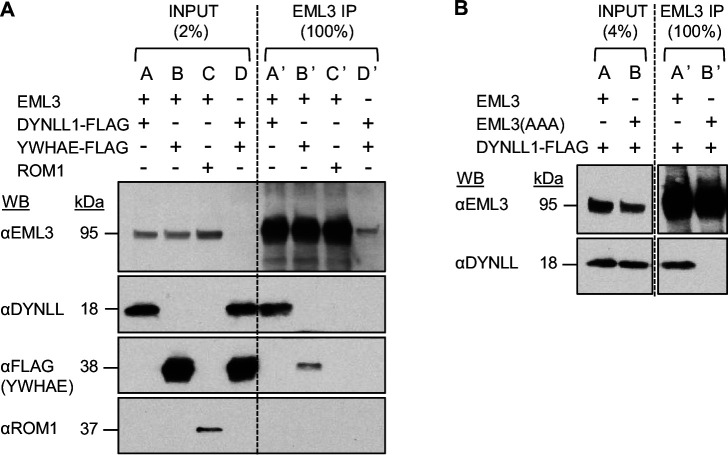
Verification of EML3 protein interactions in co-transfected cells. HEK293T cells were transfected with plasmids encoding for the indicated full-length proteins. A fraction of the cell lysates was immunoblotted for verification of protein expression (INPUT lanes, 2% or 4% of the total lysate), and the remainder was used for immunoprecipitations with EML3 rabbit polyclonal antibody (EML3 IP lanes). HEK293T cells express low amounts of endogenous EML3 protein. (**A**) EML3 interacts with DYNLL1 and YWHAE proteins in co-transfection experiments. DYNLL1 co-immunoprecipitates with co-expressed EML3 protein in lane A’. YWHAE co-immunoprecipitates with co-expressed EML3 protein in lane B’. ROM1 does not co-immunoprecipitate with co-expressed EML3 protein in lane C’. DYNLL1 and YWHAE are not immunoprecipitated by the EML3 antibody when no EML3-expressing construct is co-transfected into the cells in lane D’. (**B**) Mutating the TQT86 motif of the EML3 protein to AAA abolishes binding to DYNLL1. DYNLL1 co-immunoprecipitates with co-expressed EML3 protein in lane A’. DYNLL1 does not co-immunoprecipitate with co-expressed EML3TQT86AAA mutant protein in lane B’. Figure 7—source data 1.PDF file containing scans of original films used to prepare Figure 7A, B, indicating the relevant bands and coIP conditions. Figure 7—source data 2.Scans of original films corresponding to Figure 7A. Figure 7—source data 3.Scans of original films corresponding to Figure 7A. Figure 7—source data 4.Scans of original films corresponding to Figure 7B.

To establish the molecular mechanism by which the absence of EML3 protein leads to FNEs and intrauterine growth restriction of mouse embryos, we searched for proteins that interact with EML3. Taking advantage of *Eml3* null mice to control for non-specific interactions, we used co-immunoprecipitation coupled to mass spectrometry (coIP-MS) to find EML3-interacting proteins in vivo in tissues that could be relevant to the phenotypes that we observed. Immunoprecipitations with anti-EML3 antibody were performed on E15.5 *Eml3^+/+^* and *Eml3^−/−^* whole embryo lysates (three biological replicates per genotype) and on E12.5 *Eml3^+/+^* and *Eml3^−/−^* head lysates (two biological replicates per genotype). Additionally, as defects in extra-embryonic tissues are often associated with intrauterine growth restriction, immunoprecipitations were performed on E12.5 placenta lysates (two biological replicates). Table 3 displays the proteins that are immunoprecipitated with the EML3 antibody from each of the tissues of interest. As expected, EML3 is the most abundant protein identified in the immunoprecipitates. The most abundant co-immunoprecipitate of EML3 in E12.5 head samples is the neuron-specific tubulin beta-3 (TUBB3). Five 14-3-3 proteins were identified as co-immunoprecipitating with EML3. When all 14-3-3 spectral counts from the five paralogs are combined, the 14-3-3 protein family is the most abundant EML3 co-immunoprecipitate. Among the 14-3-3 proteins, 14-3-3 theta was the most abundant EML3 co-immunoprecipitate. EML3 contains the ideal binding site for the cytoplasmic dynein light chain (DYNLL) proteins (Rapali et al., 2011), and we identified DYNLL1 as one of the most abundant EML3 co-immunoprecipitates. The association of the EML3 protein with DYNLL1, 14-3-3 epsilon (YWHAE), and 14-3-3 gamma (YWHAG) was verified through co-immunoprecipitation in transfected cells (Figure 7 and data not shown).

**Table 3.** EML3 protein coIP-MS data from three different tissues.

				E15.5 whole embryo		E12.5 embryo head		E12.5 placenta		
				*Eml3^−/−^*	*Eml3^+/+^*	*Eml3^−/−^*	*Eml3^+/+^*	*Eml3^+/+^*	*Eml3^+/+^*	*Eml3^AAA/AAA^*
				αEML3	αEML3	αEML3	αEML3	αIgG	αEML3	αEML3
**Protein name**	**Symbol**	**UniProt ID**	**MW (kDa)**	*n* = 3	*n* = 3	*n* = 2	*n* = 2	*n* = 2	*n* = 2	*n* = 1
Echinoderm microtubule-associated protein-like 3	EML3	EMAL3_MOUSE; Q8VC03	96	0	288	0	229	1	233	80
Tubulin beta-3 chain	TUBB3	TBB3_MOUSE; Q9ERD7	50	0	0	0	50	0	0	0
14-3-3 protein theta	YWHAQ	1433T_MOUSE; P68254	28	1	8	0	0	0	24	6
14-3-3 protein epsilon	YWHAE	1433E_MOUSE; P62259	29	0	0	0	15	0	12	0
14-3-3 protein eta	YWHAH	1433F_MOUSE; P68510	28	0	0	0	0	0	26	7
14-3-3 protein zeta/delta	YWHAZ	1433Z_MOUSE; P63101	28	0	0	0	0	0	24	7
14-3-3 protein gamma	YWHAG	1433G_MOUSE; P61982	28	0	0	0	0	0	19	0
Dynein light chain 1, cytoplasmic	DYNLL1	DYL1_MOUSE; P63168	10	0	11	0	0	0	18	0
Myosin-10	MYH10	MYH10_MOUSE; Q61879	229	1	0	0	0	0	24	0
Fibrillin-2	FBN2	FBN2_MOUSE; Q61555	314	0	0	0	20	0	0	0
Gap junction alpha-1 protein	CJA1	CXA1_MOUSE; P23242	43	0	0	0	0	0	13	10
THO complex 4	ALYREF	Q0VBL5_MOUSE; O08583	27	0	13	0	0	0	0	0
60S ribosomal protein L7	RPL7	RL7_MOUSE; P14148	31	0	0	0	11	0	0	0
Integrin alpha-5	ITGA5	ITA5_MOUSE; P11688	115	0	9	0	0	0	0	0
Sfrs10 protein	TRA2B	Q5PR75_MOUSE; Q5PR75	27	0	8	0	0	0	0	0
Nucleolin	NCL	NUCL_MOUSE; P09405	77	0	0	0	7	0	0	0

To define what biological processes are dependent on an EML3–DYNLL interaction, we generated mice in which the EML3 DYNLL-binding motif was mutated to abolish binding. In EML3, the DYNLL-binding site is a TQT motif at position 86 of the protein sequence (Rapali et al., 2011). An expression construct in which the EML3 TQT motif was replaced by alanines (TQT86AAA) was generated. Figure 7B shows a co-immunoprecipitation experiment performed in cells transfected with wild-type EML3 and the TQT86AAA variant. The TQT86AAA mutation abolished EML3–DYNLL binding. A mouse line carrying the TQT86AAA mutation was generated. DYNLL was not co-immunoprecipitated with EML3 in lysates from homozygous *Eml3*^TQT86AAA^ E12.5 placentas (*Eml3^AAA/AAA^*) in coIP-MS experiments (Table 3). Remarkably, mice homozygous for the *Eml3*^TQT86AAA^ allele developed normally, with no intrauterine growth restriction and no FNEs in the developing cortex. The mutant mice survived past weaning age, appeared normal, were fertile, and had a normal lifespan without apparent health issues. Thus, the DYNLL1 binding site and EML3–DYNLL direct interaction is dispensable for EML3 functions identified in *Eml3* null mice.

## Discussion

In this study, we show that loss of EML3 results in FNEs with over-migration of neurons through a defective PBM. EML3 is the second member of the EML protein family, with EML1, whose absence leads to cortical brain malformations. However, the cortical malformation caused by the absence of EML3 contrasts with the subcortical band heterotopias observed in *Eml1* null mice (Bizzotto et al., 2017; Kielar et al., 2014).

### Intrauterine growth restriction and delayed embryo development in *Eml3* null mice

In addition to the cortical brain defect, *Eml3* null mice have a delay in embryonic development, have intrauterine growth restriction, and die perinatally. The growth restriction and somite count differences observed in *Eml3* null embryos become apparent at E8.5. No size or stage differences were seen at E7.5 nor in pre-implantation embryos. We observe no differences in staining for cell cycle (pHH3 and Cyclin B1) and apoptotic cell death (cleaved caspase 3) markers in E8.5 embryonic and extraembryonic tissues (data not shown). We did not pursue further the elucidation of the molecular mechanism responsible for the growth restriction and developmental delay of *Eml3* null embryos. Nevertheless, this delay had to be considered for the characterization of *Eml3* null FNEs. Consequently, neurodevelopmental analyses were performed on developmentally/stage-matched embryos because comparison of *Eml3* null and control littermates displayed differences in cortical development that were a consequence of the developmental delay.

### Role of EML3 in the integrity of the PBM and ECM remodeling

Our electron microscopy analyses demonstrate that the absence of EML3 results in structural defects of the PBM that precede breaching by radially migrating neuroblasts. At the TEM level, we found that the PBM of the dorsal telencephalon of *Eml3* null mice was uniformly disorganized and less dense than that of control mice (Figure 5D). However, the FNEs, which are the macroscopic structural anomaly, occur focally, are relatively few, and occur in random locations within the dorsal telencephalon (Figure 3A, C). Given that the first FNEs occurred when the first radially migrating neurons reached the already defective PBM, we propose that the abnormal PBM in *Eml3* null embryos is sporadically disrupted by the first migrating neurons of the cortical plate, creating a permanent breach through which subsequent neurons can also migrate into the adjacent subarachnoid space.

Interestingly, basement membranes other than the PBM, such as that of the skin, blood vessels, muscle, or retina, were not affected in *Eml3* null embryos (data not shown). This suggests that the absence of EML3 uniquely affects the integrity of the PBM. One possible explanation is that the component or pathway defective in *Eml3* null mice is redundant in basement membranes other than the PBM.

The small number and size of FNEs in *Eml3* null mice is comparable to the GPR56 (Li et al., 2008) and Gα_12/13_ (Moers et al., 2008) mouse models of COB. GPR56 is a receptor for the ECM component collagen III and couples to the Gα_12/13_ family of G proteins for activation of the RhoA pathway upon ligand binding (Luo et al., 2011). However, since many different receptor-ligand pairs exist, there is redundancy in the binding of ECM components to radial glial endfeet. In fact, GPR56 appears to play a lesser role in PBM formation and maintenance than other ECM receptors. For example, *Gpr56* null mice have less severe COB phenotypes than mice with mutations in dystroglycan (Geis et al., 2013; Godfrey et al., 2007; Myshrall et al., 2012; Riemersma et al., 2015). Thus, as seen for GPR56, the molecular or cellular function of the EML3 protein in PBM formation or maintenance may be partly redundant. An additional level of redundancy for EML3 function has been unveiled by transferring *Eml3* null alleles into the CD-1 outbred mouse strain. The resulting CD-1 congenic *Eml3* null mice are indistinguishable from control littermates, with no perinatal lethality, intrauterine growth restriction, nor FNEs (data not shown). Thus, CD-1 outbred mice have modifier genes that compensate for the absence of EML3, whereas inbred strains of mice 129X1 (data not shown) and C57BL/6 do not.

### Identifying the tissue(s) in which EML3 is essential for cortical brain formation

The PBM is made up of an ECM and the basal endfeet of radial glial processes. Meningeal fibroblasts in the mesenchyme contribute to the PBM by secreting and organizing most of the basement membrane constituents (Sievers et al., 1994). Cell surface receptors, such as integrins and dystroglycan, on basal endfeet of neuroepithelial cells and radial glia, orchestrate the assembly of the ECM (Schwarzbauer, 1999). Thus, the integrity of the PBM relies on both the meningeal fibroblasts and the neuroepithelial or radial glial cells of the cerebral cortex. In this study, we establish the role of EML3 in regulating PBM integrity by demonstrating that (1) loss of EML3 in mice results in defective PBM and FNEs, a cobblestone-like cortex, and (2) EML3 is expressed in both tissues that contribute to PBM formation and maintenance, namely the meningeal mesenchyme and the neuroepithelium, at the time when the PBM is forming (Figure 6). However, the tissues in which EML3 expression is essential for normal PBM formation and maintenance remain to be determined. With the conditional potential of the *Eml3^fl/fl^* mice that we have generated (Figure 1), it will be possible to delete EML3 in specific tissues and at specific developmental stages.

### Differences in the spatio-temporal expression of EML3 and EML1 proteins

Our study indicates that EML1 and EML3 proteins affect neuroblast migration in opposite directions, with EML1 deficiency resulting in subcortical band heterotopia with under-migration of neuroblasts and EML3 deficiency resulting in COB malformation with over-migration of neuroblasts. Importantly, absence of neither EML1 nor EML3 results in a neuroblast-intrinsic cell motility defect. In *Eml1* null brains, radial glia have been defined as the defective tissue, whereas in *Eml3* null brains, the two main candidate tissues are neuroepithelium and mesenchyme. Whereas the EML3 protein is present in both of the PBM forming tissues, *Eml1* mRNA was shown to be expressed in neural progenitors and not in meningeal mesenchyme (Kielar et al., 2014). The timing of the expression of EML1 and EML3 also differs, with EML3 being expressed at its highest levels until E11.5, while *Eml1* mRNA expression was observed in the radial glia, intermediate progenitors, neuroblasts, and mature neurons starting at E11.5. Tissue and developmental stage specificity in the expression of the two EML proteins may account at least in part for the contrasting effects of their absence on neuroblast radial migration.

### Analysis of microtubule-dependent cellular processes in the developing *Eml3* null brain

EML3 is a microtubule-binding protein with no documented link to PBM formation or maintenance. Identifying the cellular and molecular mechanisms by which the absence of EML3 protein results in a defective PBM may therefore unveil a novel pathological mechanism for COB.

The EML3 protein was first isolated from HeLa cell microtubule preparations (Tegha-Dunghu et al., 2008), and it was then shown to colocalize with microtubules in cell cultures (Luo et al., 2019; Tegha-Dunghu et al., 2008). Indeed, our pulldown experiments from embryo lysates supported their interaction in vivo. Moreover, we have determined that EML3 binds polymerized microtubules directly in vitro (TIRF microscopy, data not shown). Microtubule-associated proteins (MAPs) play a crucial role in regulating the dynamics and organization of microtubules in cells (Alfaro-Aco and Petry, 2015; Bodakuntla et al., 2019). The absence of EML3 protein may therefore alter microtubule dynamics or organization. However, staining of *Eml3* null and control cortices with the microtubule markers alpha-tubulin, acetylated alpha-tubulin, and tubulin beta 3 at all embryonic ages studied revealed no differences in the localization or organization of microtubules (data not shown).

Microtubules play important roles in cellular processes such as cell division, cell polarity, cell motility, and trafficking of organelles (Goodson and Jonasson, 2018; Logan and Menko, 2019). Microtubule-dependent processes were therefore considered as possible cellular mechanisms by which the absence of EML3 leads to COB. No defects in cell division were observed in the developing *Eml3* null brains. In particular, no significant differences were observed between *Eml3* null and control littermates in the mitotic index nor in the percentage of Cyclin B1 positive cells in the neuroepithelium and in the head mesenchyme in E8.5–E11.5 embryos (data not shown). Also, no differences in the angle of mitotic spindles in apical progenitors of stage-matched *Eml3* null and control embryos were observed (data not shown). We ruled out that the *Eml3* null PBM defects are a consequence of a loss of neural precursor polarity by staining neuroepithelial and radial glial cells in E9.5–11.5 *Eml3* null and control brain sections with apical and basal markers (data not shown). In areas of the *Eml3* null developing telencephalon with an intact basement membrane, neurons migrated to their appropriate destination, and the cortex is layered normally (Figure 4C). The motility of radially migrating neuroblasts therefore appears to be normal in the absence of PBM breaches. Additionally, we saw no differences in the staining pattern for ECM components between *Eml3* null and control brains (Figure 5—figure supplement 1), suggesting no major deficiencies in the secretion of main PBM ECM components. Our observations are therefore not consistent with a direct role of the EML3 protein in microtubule dynamics and organization. An alternative function for MAPs, besides a direct role in microtubule dynamics and organization, is to recruit other proteins to microtubules so they can perform their biological functions.

### EML3 protein interactions in relevant tissues and significance for FNE formation

Published high-throughput experiments using cultured cell lines have yielded many potential EML3 interacting proteins, among which the following have been observed in more than one experiment: dynein light chain proteins DYNLL1 and DYNLL2 (Hutchins et al., 2010; Huttlin et al., 2021); 14-3-3 proteins YWHAB, YWHAG, YWHAH, YWHAQ, and YWHAZ (Ewing et al., 2007; Huttlin et al., 2021); NEK kinases NEK1, NEK6, NEK7, and NEK9 (Buljan et al., 2020; Ewing et al., 2007; Golkowski et al., 2023; Huttlin et al., 2021); and beta-tubulins TUBB2A, TUBB2B, TUBB4B, TUBB8 (Huttlin et al., 2021).

As determined previously for EML1 and EML2 (Richards et al., 2014), we confirmed that EML3 binds soluble tubulin proteins. We also confirmed the interaction of EML3 with DYNLL and 14-3-3 proteins and determined with co-transfection assays that the pairwise interactions with EML3 are direct (Table 3, Figure 7, and data not shown). Moreover, using TIRF microscopy with purified proteins, we determined that EML3 can recruit DYNLL to microtubules (data not shown). Interestingly, we found that the EML3–DYNLL interaction was dispensable for the function of EML3 since the mouse line carrying the mutation in the interacting domain had no impact on mouse development.

YWHAE is the only 14-3-3 protein that we identified as an EML3 interactor in the developing brain, and *YWHAE* null mice have phenotypes similar to those of *Eml3* null mice, such as intrauterine growth restriction, perinatal death, and cortical brain malformations (Toyo-oka et al., 2003). *YWHAE* null embryos were found to have a thin cortex and slow migration of neuroblasts without inversion of cortical layers.

Luo et al. determined that EML3 regulates mitotic spindle assembly by recruiting the augmin protein complex and the gamma-tubulin ring complex to spindle microtubules (Luo et al., 2019). EML3 was shown to interact, through its C-terminal domain, with several subunits of the augmin complex as well as with gamma-tubulin. We did not co-IP any of the subunits of the augmin complex, nor gamma-tubulin, in our coIP-MS experiments. However, these interactions occur specifically during mitosis. Since we did not enrich tissue lysates for mitotic cells in our coIP-MS experiments, and mitotic cells represent ~5% of cells in these tissues, these interactions might have been below the threshold for detection. Another documented EML protein interaction that is transient and cell cycle dependent is with NEK kinases (Adib et al., 2019), and we did not identify the NEK kinases as interactors in the embryo tissues.

### Clinical relevance

Mutations in *EML1* have been linked to band heterotopia in humans (Kielar et al., 2014; Shaheen et al., 2017). To date, no *EML3* mutations of clinical relevance have been identified. Given that *Eml3* null mice are a model of COB malformation and that approximately one-third of non-syndromic COB cases have no known etiology, we analyzed a panel of DNA samples from 38 patients with COB and no known causative mutations in any of the known dystroglycanopathy genes. No *EML3* coding mutations were seen in those samples. Since COB and PMG have phenotypic and etiological similarities, we also analyzed a panel of DNA samples from 15 PMG patients and found no *EML3* coding mutations. Defining the clinical relevance for the *EML3* gene will require further testing for non-coding regulatory mutations as well as testing additional COB samples. The phenotypic spectrum for genetic analyses could also be expanded; given the phenotypes observed in our *Eml3* null mice, the *EML3* gene may have clinical implications for intrauterine growth restriction.

### Conclusion

The microtubule-binding protein EML3 is required for mammalian embryonic growth and cortical brain development. *Eml3* null mice exhibit intrauterine growth restriction with delayed embryo development and die perinatally. In addition, *Eml3* null mice have FNEs, due to neuroblasts migrating past a defective PBM into the sub-arachnoid space, phenocopying the COB malformation condition. We demonstrated that the structural integrity of the PBM is affected by the time that the first radially migrating neuroblasts reach the marginal zone of the cortical plate.

Although the EML3 protein was reported to be required for mitotic progression in cancer cell lines, we found no differences in mitotic and other cell cycle markers in *Eml3* null embryo tissues. Further cellular and molecular investigations will be necessary to deepen our understanding of how the EML3 protein controls, either directly or indirectly, neuroblast radial migration.

## Materials and methods

### Generation of Eml3 null mice

*Eml3* null mice (*Eml3*^tm1e(EUCOMM)Wtsi^) were generated from ES cell line clone EPD0751_4_D11 made by the European Conditional Mouse Mutagenesis Program (EUCOMM) and purchased from the European Mouse Mutant Cell Repository. See Skarnes et al., 2011 for the general design of the lines. Specifically, the ‘knockout-first’ allele of *Eml3* was constructed by inserting an IRES:lacZ trapping cassette and a floxed promoter-driven neo cassette into *Eml3* intron 10 based on NCBI Reference Sequence gene NM_144872. We obtained JM8A3.N1 ES cells at passage 5 (C57BL/6N mouse strain, male genotype) that were heterozygous for the targeted mutation. The ES clone contains a KO-first reporter-tagged insertion with conditional potential (allele *Eml3*^tm1a(EUCOMM)Wtsi^). The nucleotide sequence of that allele is publicly available as GenBank JN963812.

ES cells were injected into C57BL/6N blastocysts at the Goodman Cancer Center Transgenic Core Facility, and one chimeric male with germline transmission of the targeted *Eml3* allele was imported into our facility. However, our mice did not carry the distal loxP site and are thus devoid of conditional potential and therefore represent an *Eml3*^tm1e(EUCOMM)Wtsi^ allele according to EUCOMM nomenclature (Figure 1). We determined, through PCR analysis and sequencing, that the ES cell cultures contained a minor fraction (about 15%) of cells with the added loxP site within *Eml3* intron 16 in a background of cells with an intact wild-type (WT) sequence. Our founder male was therefore derived from the more prevalent cell type in the mixed ES cell population. The primers used to detect the presence of the distal loxP site were: RM173F 5′-GCAGGGAAGTCTAGGAAGGG and RM165R 5′-GGGCTGCTACCAGACTGAAG and yield amplification products of 170 bp in WT alleles or 222 bp when the distal loxP site is present. Mice were backcrossed to C57BL/6J background (Jax: 000664) for at least two generations prior to conducting experiments and have currently been backcrossed for eight generations. The C57BL/6N-derived retinal degeneration *Crb1^rd8^* spontaneous mutation was eliminated from our mice by selecting *Crb1^wt/wt^* progeny at the N2 generation. Experimental mice were produced by intercrossing heterozygotes.

We then proceeded to generate conditional *Eml3* null mice (*Eml3^fl/fl^*) in a stepwise process starting from the constitutive *Eml3* null (*Eml3*^tm1e(EUCOMM)Wtsi^) mice described above. The first step was to introduce a distal loxP site for Cre-directed removal of coding exons. We opted for a CRISPR–Cas9 mediated homology-directed repair (HDR) technique to introduce a loxP site into *Eml3* intron 19 based on NCBI Reference Sequence gene NM_144872 (genomic position 8,940,293 bp according to GRCm38/mm10 assembly). gRNA 5′-ACTGTTAGTGCTTTATCCAGAGG was used in conjunction with the asymmetric reverse strand repair template (single-stranded oligo deoxynucleotides; ssODN) 5′-TGGGGGCCACCCTGGTCTACAAACGCAGGTGACAGAGGGAACCCTGTCTCAGAAACAGAGAATGTAGCCTTAGACATGCCTAGAGcctctgataacttcgtataatgtatgctatacgaagttatgataaagcactaacagtTTCTCATCAGCTGCCCCTC. The CRISPR-Cas9 reagents: 50 ng/µl cas9 mRNA (Sigma-Aldrich, CAS9MRNA-1EA), 20 ng/µl in vitro transcribed gRNA, and 100 ng/µl ssODN, were microinjected into the cytoplasm of 1-cell stage embryos obtained from in vitro fertilization of C57BL/6N females with sperm from heterozygous *Eml3*^tm1e(EUCOMM)Wtsi^ males. One chimeric male was obtained with in-cis insertion of the distal loxP site into the *Eml3*^tm1e(EUCOMM)Wtsi^ allele. Presence of the intron 19 loxP site was detected by PCR amplification with primers flanking the insertion site and yielding an amplification product of 271 bp in the WT allele or 305 bp when the distal loxP site is present: RM226F 5′-CCGGCTGCACCAAGAAG and RM227R 5′-TTCTCACTGACTTGGCTTGG. The PCR products were sequenced for confirmation that the inserted loxP site was intact. In-cis insertion of the distal loxP site with the *Eml3*^tm1e(EUCOMM)Wtsi^ allele was verified by in vitro recombination with Cre enzyme (NEB Cat#M0298M) followed by diagnostic PCRs with primers flanking the proximal loxP site within *Eml3* intron 10 (RM177F 5′-AAAACCTCCCACACCTCCC) and distal loxP site within *Eml3* intron 19 (RM227R 5′-TTCTCACTGACTTGGCTTGG), yielding an amplification product of 414 bp only when the distal loxP site was inserted in-cis with the proximal loxP site and only in the presence of Cre recombinase. Mice were backcrossed to C57BL/6J background (Jax: 000664) for at least two generations, and the C57BL/6N-derived retinal degeneration *Crb1^rd8^* spontaneous mutation was eliminated from our mice by selecting *Crb1^wt/wt^* progeny at the N2 generation. The resulting mouse, *Eml3^em1Mci^*, is a gene trap-floxed allele ‘*Eml3^gt-fl^*’, (Figure 1C) and is phenotypically indistinguishable from *Eml3^tm1e(EUCOMM)Wtsi^* mice. The second step was to excise the Gene Trapping cassette and thus produce a WT *Eml3* allele with conditional potential (floxed exons 11–19; *Eml3^em1.1Mci^*) that is a floxed allele ‘*Eml3^fl^*’ (Figure 1D). Trap-floxed allele *Eml3^gt-fl^* mice were crossed to the Flp1 line (B6.129S4-*Gt(ROSA)^26Sortm1(FLP1)Dym^*/RainJ; Jax: 009086) to remove the gene trap, lacZ reporter, and neomycin selection cassettes between the two FRT sites within intron 10 of the *Eml3* gene. The FLP recombinase gene was then eliminated in the following breeding history.

Exposure to Cre recombinase removes exons 11–19 leading to a frameshift and premature stop codon, which is predicted to lead to nonsense mediated decay of the mRNA. Absence of the EML3 protein was confirmed by immunoblotting and indirect immunofluorescence. Crossing *Eml3^fl/fl^* mice with hemizygous B6.FVB*-Tg(EIIa-cre)^C5379Lmgd^*/J mice (JAX: 003724) resulted in germline recombination of the loxP sites and therefore, after removal of the *E2a-Cre* transgene in the following breeding history, an *Eml3* null allele that does not rely on the activity of a gene trap cassette. This new global knockout mouse is named *Eml3^em1.2Mci^* (Figure 1E). Mice were backcrossed to C57BL/6J background (Jax: 000664) for at least two generations prior to conducting experiments and have currently been backcrossed for six generations. Experimental mice were produced by intercrossing heterozygotes. These mice have been determined to be phenotypically indistinguishable from *Eml3*^tm1e(EUCOMM)Wtsi^ and from *Eml3^em1Mci^* mice.

We generated a number of tissue-specific *Eml3*-deficient mice by crossing our *Eml3^fl/fl^* mice with different mouse lines expressing the cre recombinase under the control of promoters that are active in cells and at developmental stages with potential relevance to the phenotypes observed in the global KO. Sox1, Mesp1, and Pdgfra cre drivers were used to delete *Eml3* in tissues involved in development of the PBM. F2 crosses of *Eml3^fl/fl^* mice with hemizygous B6.B6CB-Sox1tm1(cre)Take mice (Sox1-Cre; Riken BRC no. RBRC05065) resulted in *Eml3* deletion specifically in neuroepithelium. ICR.Cg-Mesp1tm2(cre)Ysa/YsaRbrc mice (Mesp1-Cre; Riken BRC no. RBRC01145) were first backcrossed a minimum of five times into the B6J genetic background before being used to delete *Eml3* specifically in mesoderm-derived mesenchyme. B6.Cg-E2fltg(Wnt1-Cre)2Sor/J mice (Wnt1-Cre; JAX: 022501) and B6-Tg(Pdgfra-cre)1Clc/J mice (Pdgfra-Cre; JAX: 013148) were used to delete *Eml3* specifically from neural crest-derived mesenchyme. All three tissue-specific nulls were indistinguishable from *Eml3^wt^* controls. *Sox1*, *Mesp1*, and *Pdgfra* cre driver pairs and the combination of all three were also indistinguishable from *Eml3^wt^* controls. We also used B6.FVB-Tg(Ada*-cre)5Xiay/J cre driver mice (JAX: 036543) that resulted in *Eml3* deletion in placenta. In all cases, despite confirmed tissue-specific absence of the EML3 protein, the resulting mice were phenotypically indistinguishable from *Eml3^wt^* controls.

### Genotyping of Eml3 null mice

Two PCR genotyping strategies were used in parallel for unequivocal genotype determination.

A four-primer PCR strategy yields a 286-bp PCR product for the wild-type *Eml3* allele (*Eml3^wt^*), a 792-bp PCR product for both the gene trap allele (*Eml3^gt^*) and the gene trap-floxed allele (*Eml3^gt-fl^*), a 550-bp PCR product for the floxed allele (*Eml3^fl^*), and a 380-bp PCR product for the tissue-specific (*Eml3^fl^;Cre*) and the global (*Eml3^em1.2Mci^*) *Eml3-*null alleles. The four-primer *Eml3* genotyping was done using a touchdown PCR technique with the common reverse primer RM170 5′-ACACCAGGGTGGTCTCAAAC within *Eml3* intron 10, distal to the insertion site for the targeting cassette, and the forward primers RM070 5′-TGATGTTCTAGGCAGGATGTTC within *Eml3* intron 10, proximal to the insertion site for the targeting cassette, or RM169 5′-CGCCTTCTATGAAAGGTTGG within the inserted Neo cassette, for the wild-type (286 bp) or mutant (792 bp) allele amplifications, respectively. The RM070-RM170 primer pair yields a 550-bp product for the floxed allele (*Eml3^fl^*). The additional reverse primer RM227 5′-TTCTCACTGACTTGGCTTGG within *Eml3* intron 19, distal to the inserted loxP site, yields a 380-bp PCR product in combination with the forward primer RM070 for exon-deleted tissue-specific (*Eml3^fl^;Cre*) and global (*Eml3^em1.2Mci^*) *Eml3-*null alleles.

A three-primer PCR strategy yields a 521-bp PCR product for the *w*ild-type *Eml3* allele (*Eml3^wt^*), a 446-bp PCR product for both the gene trap allele (*Eml3^gt^*) and for the gene trap-floxed allele (*Eml3^gt-fl^*), and a 786-bp PCR product for the floxed allele (*Eml3^fl^*). The tissue-specific (*Eml3^fl^;Cre*) and the global (*Eml3^em1.2Mci^*) *Eml3-*null alleles do not yield a PCR product with this strategy. The three-primer *Eml3* genotyping was done using a touchdown PCR technique with the common forward primer RM070 5′-TGATGTTCTAGGCAGGATGTTC within *Eml3* intron 10, proximal to the insertion site for the targeting cassette, and the reverse primers RM071 5′-CCCACTGGTGACAATACAGC within *Eml3* exon 11 or RM075 5′-GTACCCCAGGCTTCACTGAG within the proximal inserted FRT site and the GeneTrap cassette, for the wild-type (521 bp) or mutant (446 bp) allele amplifications, respectively.

### Generation and characterization of Eml3 TQT86AAA mice

*Eml3* TQT86AAA mice (*Eml3*^AAA/AAA^) were generated at The Center for Phenogenomics in Toronto, Canada. A CRISPR-Cas9 mediated HDR technique was used to introduce nucleotide changes c.256_262ACCCAAA >gCCgcAg based on NCBI Reference Sequence gene NM_144872. gRNA 5′-GGTGACCAGAGGCACCCAAA was used in conjunction with the asymmetric reverse strand repair template (single-stranded oligo deoxynucleotides; ssODN) 5′-AGAGCTGGAGGACCATTGCTCAGGCCAGGGGGTCCAGAGGATGGAACAATCTCCAATTCTTCTTCCGcTgcGGcGCCTCTGGTCACCAAGGAAGGGCTGCATGTAGGTGGCAGTCCTGGAGGGACTGTGATGCTGCTGAG. The CRISPR–Cas9 reagents were microinjected into the cytoplasm of 1-cell stage C57BL/6J embryos. Founder chimeric mice carrying the TQT86AAA allele were identified by PCR amplification with primers flanking the targeted site and yielding an amplification product of 542 bp: RM055F 5′-GGGTGCAGGAAGAAGAGATG and RM067R 5′- TGGTGGGACAGAATGAAAAAG. The PCR products were sequenced. *Eml3* TQT86AAA mice were backcrossed to C57BL/6J background (Jax: 000664) for at least two generations. Routine genotyping was carried out with the same PCR amplification, followed by *Tau*I restriction digests that yield 477, 36, and 29 bp bands in the WT allele and 280, 197, 36, and 29 bp bands in the mutant allele. These mice were determined to be phenotypically undistinguishable from *Eml3^wt^* mice.

### Mice

Breeding animals were on a 12 hr light/dark schedule. Food and water were available ad libitum. Timed pregnant females (E7.5–E18.5) were sacrificed by CO_2_ asphyxiation after isoflurane anesthesia, followed by cervical dislocation. Ages of mice analyzed are given as embryonic day (E), where the presence of a vaginal plug was considered as embryonic day E0.5. Embryos were dissected from the uteri and placed in PBS on ice. Weight of embryos and placentas was measured in an analytical balance after drying off excess fluid. Embryo sizes were measured as area of the section corresponding to the sagittally bisected E8.5–E10.5 embryos or as weights for E11.5–E18.5 embryos. All experiments on adult mice were performed using both male and female littermates, and all data shown is pooled from both sexes after verification that there were no sex-dependent differences. Animal care was in accordance with the federal Canadian Council on Animal Care, as practiced by McGill University and the Lady Davis Research Institute.

### Immunoblotting of E15.5 whole embryo lysates

E15.5 whole embryo lysates were prepared under conditions that are compatible with downstream immunoblotting as well as with coIP-MS experiments. Uteri containing E15.5 embryos were dissected out of euthanized pregnant females and placed in a petri dish filled with PBS on ice. Each conceptus was dissected out of the uterus and transferred to a fresh petri with PBS to dissect the embryo out of the amniotic sac. The tip of the tail was collected for genotyping. The embryo was then placed in 1 ml of cold 0.5% NP-40-containing lysis buffer in a 5-ml round bottom tube (20 mM Tris-HCl, pH 8.0; 200 mM NaCl; 1 mM EDTA; 0.5% NP-40; protease inhibitors Aprotinin, Leupeptin, Pepstatin A, PMSF; 1 mM Na_3_VO_4_; phosphatase inhibitors NaF, NaPPi, beta-glycerophosphate). The embryo was then homogenized with two 10-s pulses using a polytron microtip. The homogenate was decanted into a 1.5-ml tube and rotated for 2 hr at 4°C. The lysates were then cleared by centrifugation at 21 K RCF for 15 min at 4°C. The supernatant (1.2 ml) was aliquoted into fresh tubes and conserved at –80°C. Protein quantification was performed using a microvolume BCA assay normalized against BSA standards.

50 µg of each embryo lysate was resolved on denaturing SDS–PAGE gels (4–20% gradient TGX precast gels, Bio-Rad) and transferred onto nitrocellulose membranes (Amersham Protran supported membrane, GE Healthcare) using a Mini-PROTEAN electrophoresis apparatus (Bio-Rad). Membranes were blocked with 5% milk in TBST buffer and incubated with primary antibodies overnight at 4°C and then HRP-conjugated secondary antibodies for 30 min at room temperature. The immunoreactivity of proteins was visualized with Amersham ECL Western Blotting Select Detection Reagent (GE Healthcare) using a ChemiDoc Imager (Bio-Rad). GAPDH immunoreactivity was used as loading control.

### Antibodies

Rabbit antibodies to EML3 were raised by injecting rabbits with a KLH-conjugated peptide encoding the C-terminal tail (884–897) of EML3 (Pierce/Thermo Fisher). Crude serum was used at a 1:1000 (vol/vol) dilution for immunoblotting. Affinity-purified antibodies were isolated from antisera and used at 1:25 (vol/vol) in immunofluorescence labeling. Rabbit monoclonal anti-GAPDH was used as a loading control for immunoblots (NEB 5174P; 1:4000). HRP-conjugated secondary antibodies were used for immunoblotting: donkey polyclonal anti-rabbit IgG (Amersham NA934) and sheep polyclonal anti-mouse IgG (Amersham NA931), both used at 1:20,000 (vol/vol) dilution.

The following primary antibodies were also used for immunofluorescence (dilutions in vol/vol): rabbit anti-CCNB1 (G2/M-specific cyclin-B1) (NEB/Cell signaling 4138; 1:200), goat anti-collagen IV (Southern Biotech 1340-01; 1:100), rat anti-CTIP2 (chicken ovalbumin upstream promoter transcription factor-interacting protein 2) (Abcam ab18465; 1:500), rabbit anti-CUX1 (Homeobox protein cut-like 1) (Santa Cruz sc-13024; 1:50), mouse anti-glycosylated α-dystroglycan antibody (DSHB U. Iowa IIH6 C4; 1:50), rat anti-integrin α6 (Millipore MAB1378MI; 1:100), rabbit anti-Engelbreth-Holm-Swarm laminin (Millipore-Sigma L9393; 1:500), rabbit anti-PAX6 (Paired box protein Pax-6) (Covance PRB-278P; 1:500), rabbit anti-pHH3 (phospho-Histone H3) (Millipore Sigma H0412; 1:200), mouse anti-pHH3 (phospho-Histone H3) (NEB/Cell signaling 9706; 1:200), goat anti-SOX1 (Transcription factor SOX-1) (Novus AF3369; 1:40), rabbit anti-TBR1 (T-box brain protein 1) (Abcam ab31940; 1:5000), rabbit anti-TBR2 (Eomesodermin homolog/T-box brain protein 2) (Abcam ab23345; 1:500), rabbit anti-TLE4 (Transducin-like enhancer protein 4) (kind gift from Dr. S. Stifani, McGill University, Montreal; 1:500), and mouse anti-TUBB3 (Tubulin beta-3) (Promega PR-G7121; 1:1000).

The following secondary antibodies from Invitrogen were used at a 1:500 (vol/vol) dilution for immunofluorescence: donkey anti-goat AF594 A-11058, donkey anti-mouse AF405 A-48257, donkey anti-mouse AF594 A-21203, donkey anti-mouse AF647 A-31571, donkey anti-rabbit AF488 A-21206, goat anti-rabbit AF660 A-21073, and donkey anti-rat AF488 A-21208.

The following primary antibodies were also used for immunoblotting: rabbit anti-DYNLL (Dynein light chain LC8) (Origene TA303752; 1:2,000), mouse anti-FLAG (Origene TA50011-100; 1:4000), and mouse anti-ROM1 (Rod outer segment membrane protein 1) (kind gift from Dr. R. Molday, UBC, Vancouver; 1:20).

### Tissue preparation, histology, and immunofluorescence labeling

Timed embryos were harvested after isoflurane anesthesia, CO_2_ asphyxiation, and cervical dislocation of pregnant dams.

Embryo heads (E9.5–E18.5) were fixed at 4°C using 2% paraformaldehyde-lysine-periodate in PBS and cryoprotected in graded sucrose concentrations (10%, 20%, and 30% sucrose-PBS), frozen in OCT (optimal cutting temperature) compound (Tissue Plus, Fisher HealthCare) over dry ice and sectioned on a cryostat. Cryosections (12–16 µm) were collected on Superfrost plus slides and were either stained with 0.5% cresyl violet/0.3% acetic acid (Nissl staining) or processed for immunofluorescence labeling, as described below.

Cryosections were blocked in Mouse-on-Mouse blocking solution for at least 1 hr. Sections were then incubated in primary antibodies, diluted in Mouse-on-Mouse antibody diluent, for 1 hr at room temperature. Following washes with HBS-Triton X-100, primary antibodies were visualized by incubating for 30 min at RT with appropriate fluorophore-conjugated secondary antibodies (Invitrogen; 1:500). For double or triple labeling, antibodies were applied concurrently during primary and secondary antibody steps. Washed sections were counterstained with Hoechst 33258 (Sigma) and mounted with coverslips and Fluoromount-G (Southern Biotech). All images were captured using a Quorum Wave FX SD confocal microscope (Leica) and processed using the Volocity software package or FIJI. Representative photographs were obtained with the same exposure setting for control and *Eml3* null.

For E7.5 and E8.5 embryos, following euthanasia of timed-pregnant female mice, each conceptus was isolated from the uterus and fixed in 4% paraformaldehyde-PBS for 30 min at 4°C. After rinses in PBS, the conceptus was then cryopreserved in 10%, 20%, and 30% sucrose-PBS before embedding in OCT compound. Cryosections were processed as for embryo heads.

For whole brain immunolabeling at E15.5, embryo heads were first degloved and then sectioned horizontally below palate to keep brain intact in cranium. Crania were fixed in DMSO:methanol at –20°C, then rehydrated for permeabilization in 1% Triton X-100, followed by blocking in BSA, and then primary antibody incubation, which was carried out for 5 days at 4°C. Following washes in blocking solution, crania were incubated with secondary antibodies (Invitrogen; 1:200) for 2 days at 4°C. After further washes in blocking solution, crania were dehydrated in graded methanol solutions and finally cleared in benzyl alcohol:benzyl benzoate (BABB) for visualization under a fluorescence stereomicroscope (Zeiss). Smaller regions of the dorsal telencephalon/PBM were then also imaged on a Quorum Wave FX SD confocal microscope (Leica).

### Electron microscopy

Embryo heads obtained from intercrosses of *Eml3* heterozygous mice were dissected and fixed by immersion in 2.5% glutaraldehyde in 0.1 M sodium cacodylate buffer, pH 7.4. After fixation for 24–48 hr, tissues were postfixed in 1% osmium tetroxide in cacodylate buffer, then dehydrated and embedded in Embed 812 resin (EMS). Ultrathin sections were cut and stained with uranyl acetate and lead citrate. The samples were observed and imaged using the transmission electron microscope Tecnai G2 Spirit BioTWIN fitted with a Gatan Ultrascan 4000 camera, at the McGill Facility for Electron Microscopy. For the 31–33 sp stage, four biological replicates for each genotype were analyzed. For the 39–41 sp and the 46–47 sp stages, two biological replicates for each genotype were analyzed at each timepoint.

### coIP-MS in mouse lysates

Immunoprecipitations were performed on E15.5 *Eml3^+/+^* and *Eml3^−/−^* whole embryo lysates (three biological replicates per genotype). Lysates were prepared as described for immunoblotting, using a 0.5% NP-40 detergent lysis buffer with protease and phosphatase inhibitors. Immunoprecipitations were also performed on E12.5 *Eml3^+/+^* and *Eml3^−/−^* head lysates (two biological replicates per genotype) and on E12.5 placenta lysates (two biological replicates). E12.5 tissue lysates were prepared with the following changes to accommodate the small tissues. The tissues were processed in a 420-µl volume of tissue lysis buffer and were disrupted with a sonicator using five three-second pulses at 40-s intervals. The final tissue lysate volumes were approximately 500 µl each.

Co-immunoprecipitations were performed as follows. 3 µg of rabbit polyclonal anti-EML3 C-terminal antibody (affinity purified) was bound to 20 µl of washed anti-rabbit IgG Dynabeads (Invitrogen) in PBST buffer for 1 hr at 4°C with rotation. The anti-EML3 bound beads were then added to 1.5 mg of tissue lysate for a total constant volume of 750 µl adjusted with tissue lysis buffer. The bead–antibody–lysate mixture was rotated overnight at 4°C. The beads were then washed four times for 20 min each at 4°C with rotation in a wash buffer (50 mM Tris-HCl, pH 8.0; 150 mM NaCl; 5 mM EDTA; 0.1% NP-40; 1:1000 vol/vol of each of the protease inhibitors Aprotinin, Leupeptin, Pepstatin A, PMSF; 1 mM Na_3_VO_4_; 1:100 vol/vol of each of the phosphatase inhibitors NaF, NaPPi, beta-glycerophosphate). The beads were then washed five times for 30 min each at 4°C with rotation in fresh 50 mM Ammonium Bicarbonate buffer. The beads were finally suspended in a 50-µl volume of Ammonium Bicarbonate buffer and frozen at –80°C. The beads were then processed for mass spectrometry at the IRIC proteomics platform. Processing included on-bead Trypsin digestion before loading on mass spectrophotometer.

*Eml3* null tissues served as controls for EML3 coIP specificity. However, since the placenta of *Eml3^−/−^* embryos contains EML3-expressing tissues derived from the *Eml3*^+/−^ mother (data not shown), a control IgG antibody was used on *Eml3*^+/+^ placenta lysates to control for non-specific interactions in those immunoprecipitations. Following mass spectrometry of the digested immunoprecipitates, the spectra obtained for each sample were assigned to peptides and ultimately to proteins. For each protein, the total number of spectra identified in each tissue is shown. For inclusion in the list, a lower cut-off limit of five spectra when all three tissue datasets are combined was used. A minimal enrichment of at least ten-fold in *Eml3*^+/+^ vs either *Eml3*^−/−^ or IgG control samples was used as a criterion for inclusion in the list.

### coIP of proteins overexpressed in cultured cells

The association of the EML3 protein with DYNLL1, 14-3-3 epsilon (YWHAE), and 14-3-3 gamma (YWHAG) was verified through co-immunoprecipitation in transfected cells. HEK293T cell monolayers were transfected with mammalian expression constructs using JetPrime transfection reagent (Polyplus) according to manufacturer instructions. The expression constructs were purchased from Origene (Rockville, MD) and contained mouse cDNA inserts for untagged *Eml3* (MC201737), *Dynll1*-FLAG (MR219424), *Ywhae*-FLAG (MR203269), untagged *Rom1* (MC205489), or the custom made *Eml3^TQT86AAA^* construct (MC201737 MUTANT). The expression vectors used were pCMV6-Kan/Neo for untagged constructs and pCMV6-Entry for Myc-DDK/FLAG tagged constructs. After 48 hr, the cells were washed three times with ice-cold PBS and then lysed by adding 1 ml of ice-cold IP lysis buffer (50 mM Tris-HCl, pH 7.4; 150 mM NaCl; 1 mM EDTA; 1% NP-40; protease inhibitors Aprotinin, Leupeptin, Pepstatin A, PMSF; 1 mM Na_3_VO_4_; phosphatase inhibitors NaF, NaPPi, beta-glycerophosphate). The lysate was transferred into 1.5 ml microtubes. After an incubation on ice for 30 min, the lysates were cleared by centrifugation at 21 K RCF for 15 min. The supernatants were then aliquoted into new tubes. The total yield is typically 1.5 mg of protein in a 1.25-ml volume.

2 µg of affinity purified EML3 antibody was added to 300 µl aliquots of the transfected cell lysates and incubated overnight at 4°C with rotation. 80 µl of washed anti-rabbit IgG Dynabeads (Invitrogen) were then added to the immunoprecipitation samples and rotated for 3 hr at 4°C. The bead complexes were then washed three times in a Ca^2+^ and Mg^2+^ free PBS supplemented with 0.1% BSA and 2 mM EDTA, pH 7.4. The bead complexes were then resuspended in 12 µL of 2X sample loading buffer and boiled for 5 min. The eluates (immunoprecipitates) were then loaded on denaturing SDS–PAGE gels (4–20% gradient TGX precast gels, Bio-Rad) in parallel with small aliquots from the original cell lysates (input). Immunoblotting was performed as indicated above.

### Statistical analysis

Statistical analysis was performed using GraphPad Prism. Data are shown as mean ± SD or as mean of means ± SEM. The variance was estimated for each set of data and the significance test was adapted accordingly. All tests were two-sided. Comparisons of means in two groups were made using either an unpaired Student’s *t*-test or with a Welch’s *t*-test to correct for unequal variances. Comparisons of means in three groups were made using non-parametric one-way ANOVA followed by Dunn’s pairwise tests.

## Data Availability

Source data files have been provided.

## Additional files

MDAR checklist

Reporting standard 1. ARRIVE checklist.
