# Supplementary material for: The microtubule-binding protein EML3 is required for mammalian embryonic growth and cerebral cortical development, and Eml3 null mice are a model of cobblestone brain malformation
Source: eLife. 2026 Jul 9;14:RP107102. doi: 10.7554/eLife.107102 (PMC13349382; doi:10.7554/eLife.107102)
Supplement: Figure 1—source data 1. [file elife-107102-fig1-data1.zip › Figure 1-source data 1/Figure 1-Source data 1.pptx]

## Slide 1
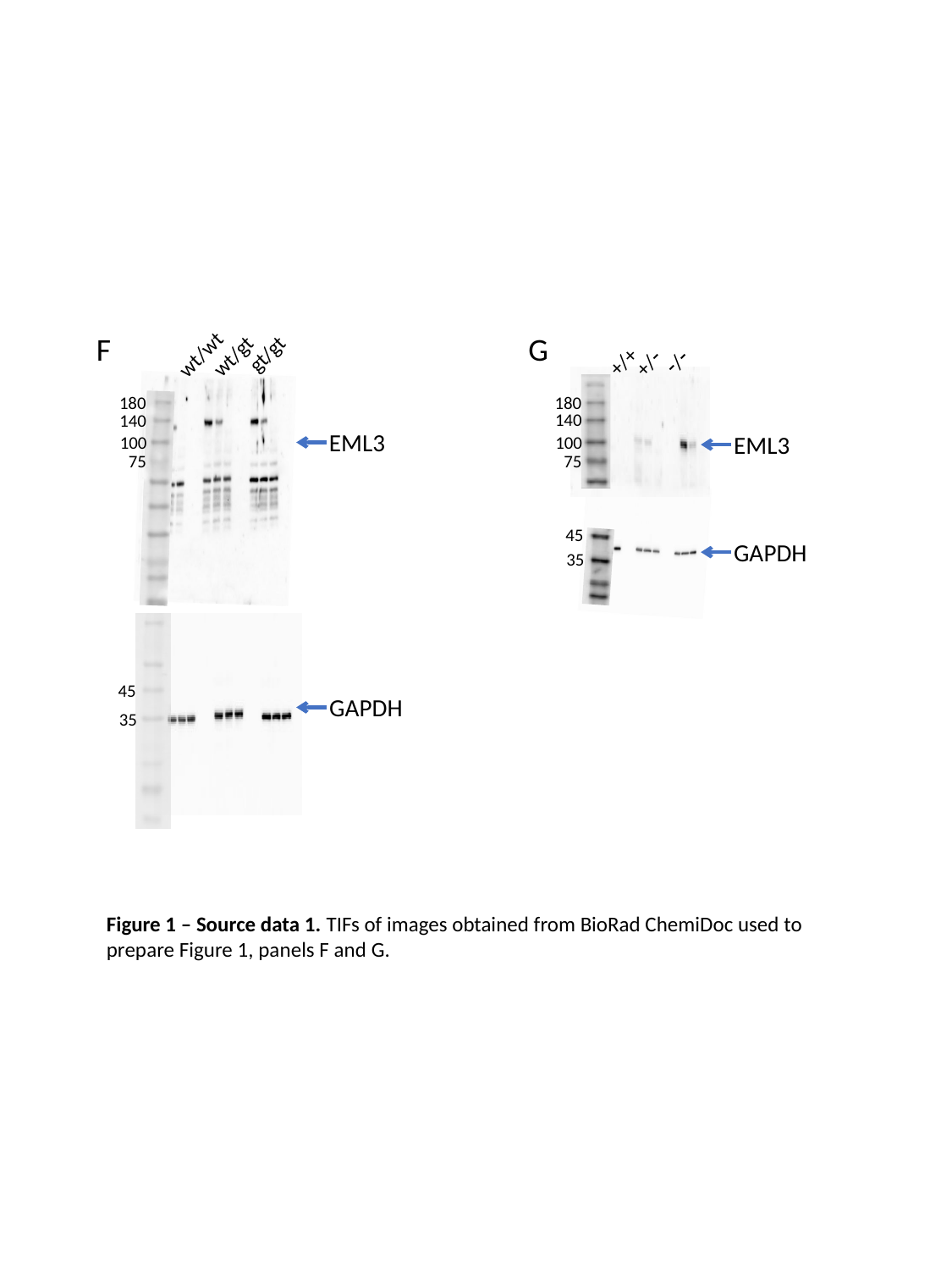

F
G
-/-
+/-
+/+
180
140
EML3
100
75
45
GAPDH
35
gt/gt
wt/wt
wt/gt
180
140
EML3
100
75
45
GAPDH
35
Figure 1 – Source data 1. TIFs of images obtained from BioRad ChemiDoc used to prepare Figure 1, panels F and G.
