# Supplementary figures and images for: The microtubule-binding protein EML3 is required for mammalian embryonic growth and cerebral cortical development, and Eml3 null mice are a model of cobblestone brain malformation
Source: eLife. 2026 Jul 9;14:RP107102. doi: 10.7554/eLife.107102 (PMC13349382; doi:10.7554/eLife.107102)

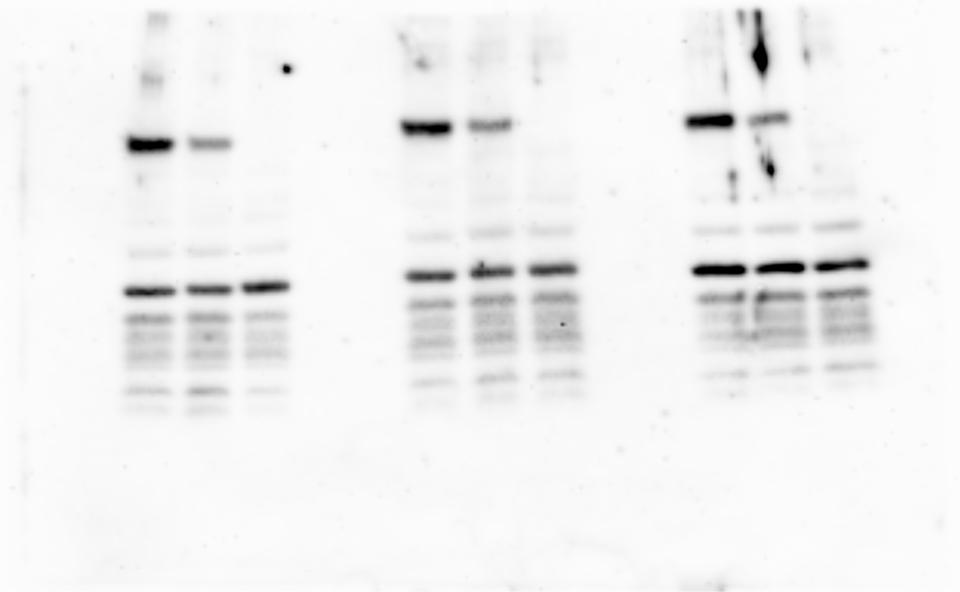

Supplement: Figure 1—source data 2. [file elife-107102-fig1-data2.zip › Figure 1-source data 2/Tm1e EML3 4min 6x6 expo.tif]

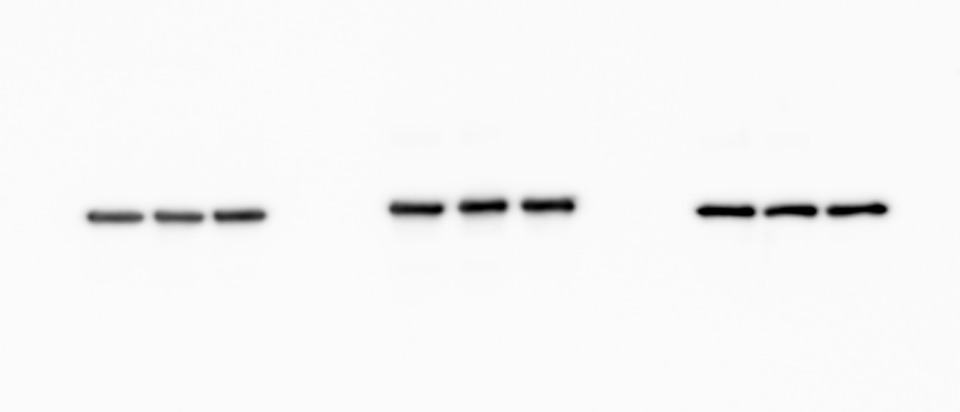

Supplement: Figure 1—source data 2. [file elife-107102-fig1-data2.zip › Figure 1-source data 2/Tm1e GAPDH 27s 4x4 expo.tif]

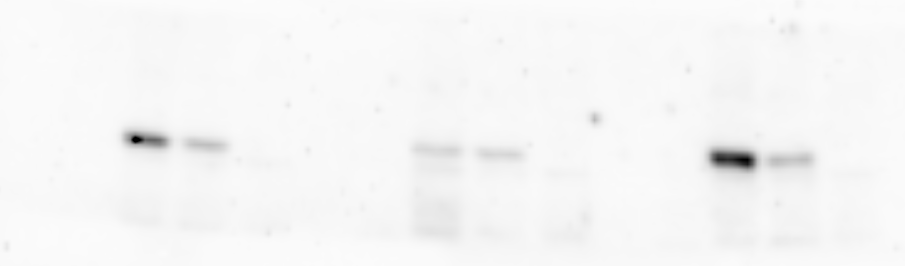

Supplement: Figure 1—source data 2. [file elife-107102-fig1-data2.zip › Figure 1-source data 2/Tm2d EML3 5min 8x8 expo.tif]

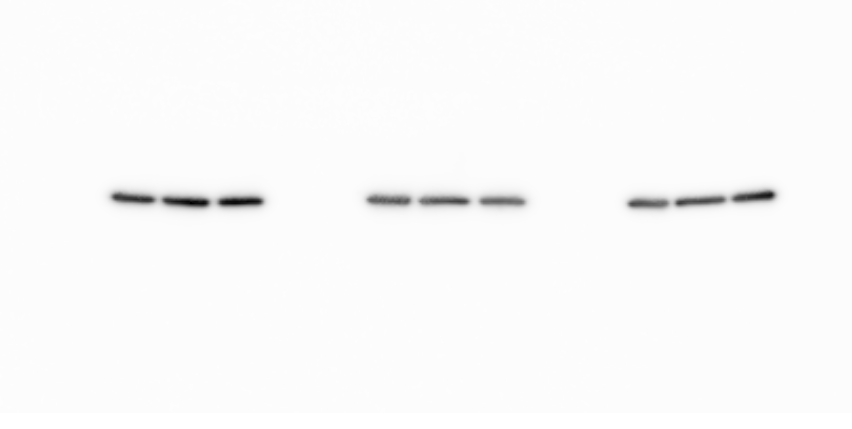

Supplement: Figure 1—source data 2. [file elife-107102-fig1-data2.zip › Figure 1-source data 2/Tm2d GAPDH 12s 4x4 expo.tif]

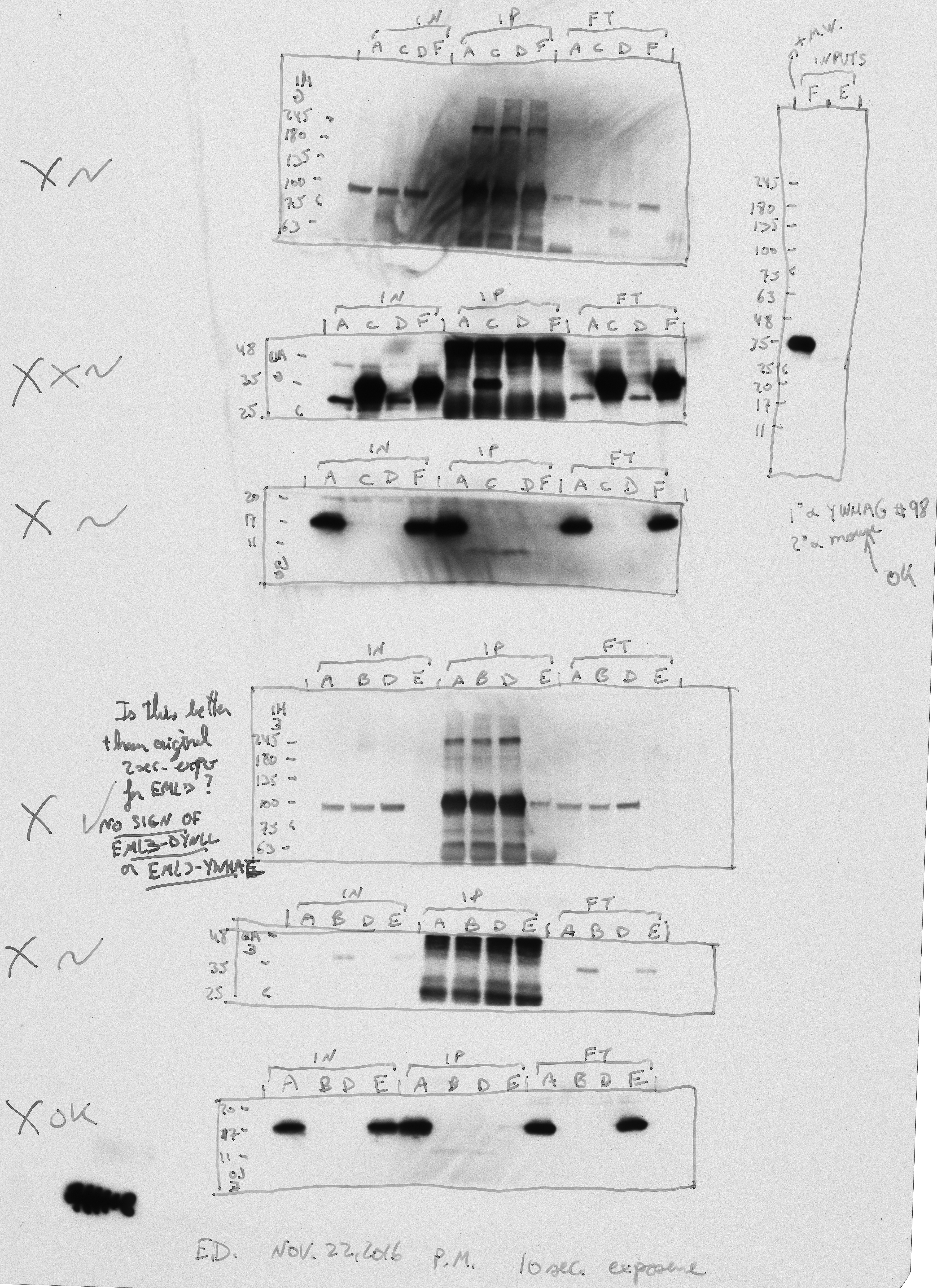

Supplement: Figure 7—source data 2. [file elife-107102-fig7-data2.zip › Figure 7A_1-source data 2/EML3 YWHA coIP 10sec expo - EML3.tif]

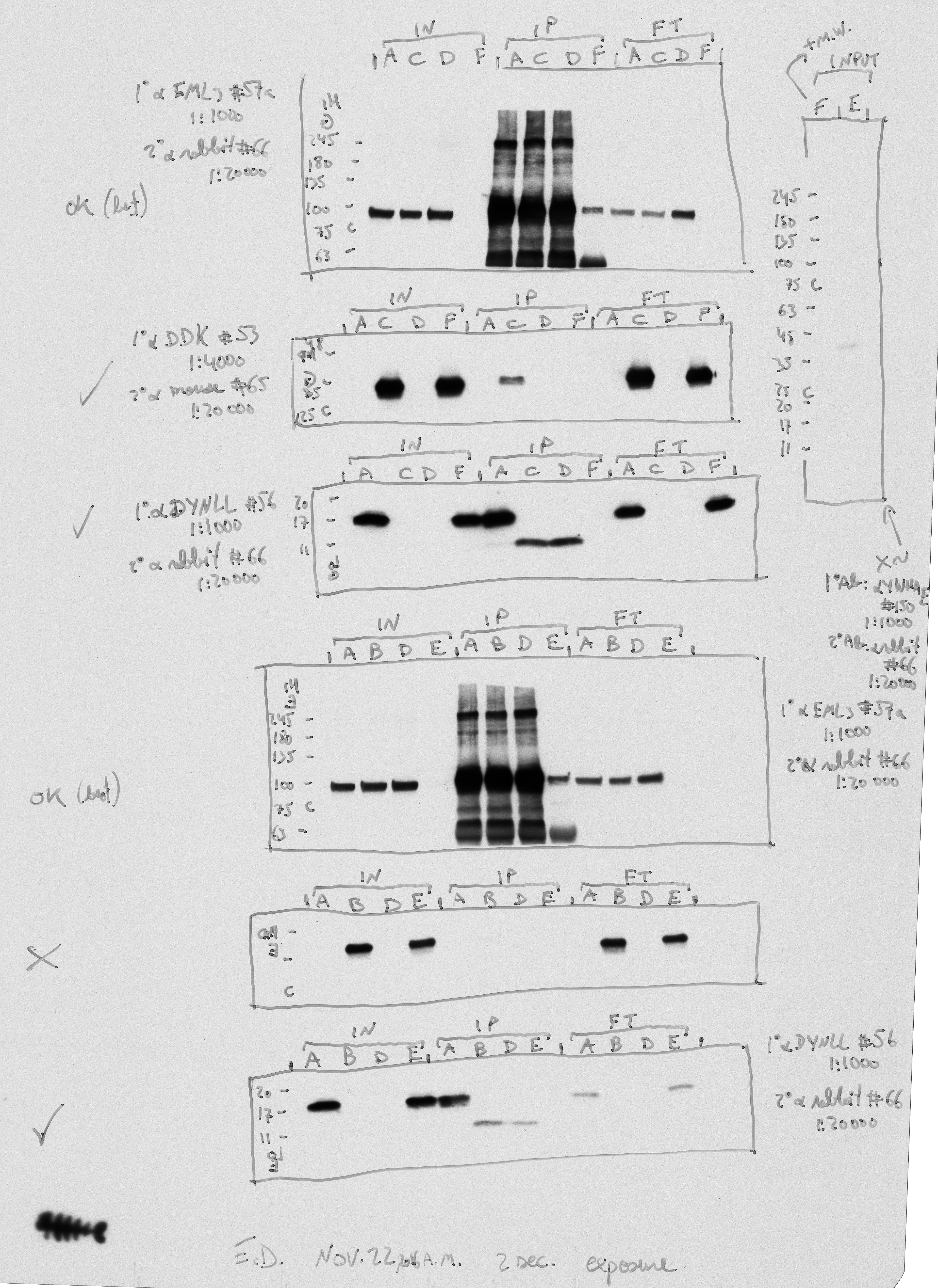

Supplement: Figure 7—source data 2. [file elife-107102-fig7-data2.zip › Figure 7A_1-source data 2/EML3 YWHA coIP 2sec expo - DYNLL1.tif]

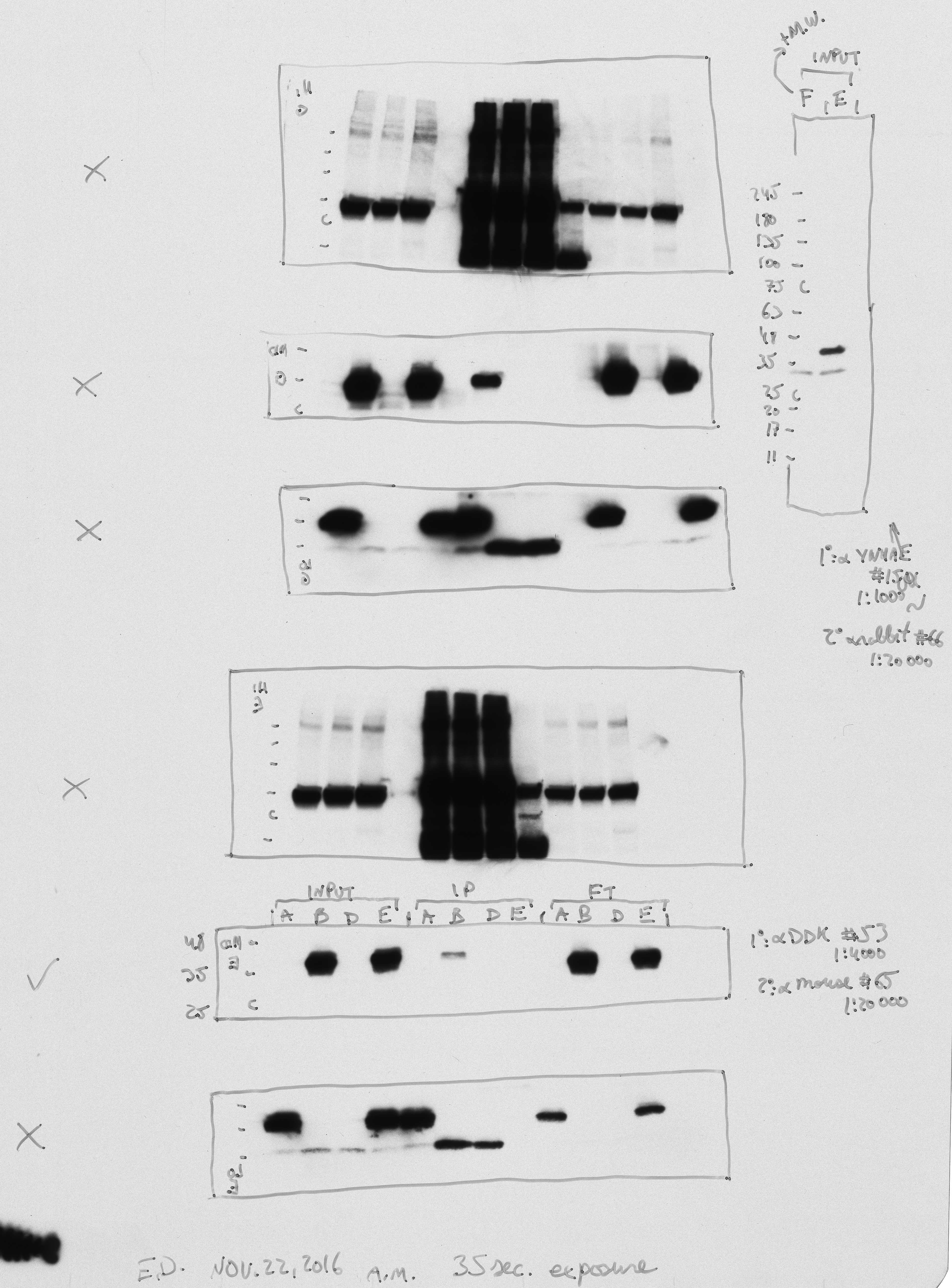

Supplement: Figure 7—source data 3. [file elife-107102-fig7-data3.zip › Figure 7A_2-source data 2/EML3 YWHA coIP 35sec expo - FLAG-YWHAE.tif]

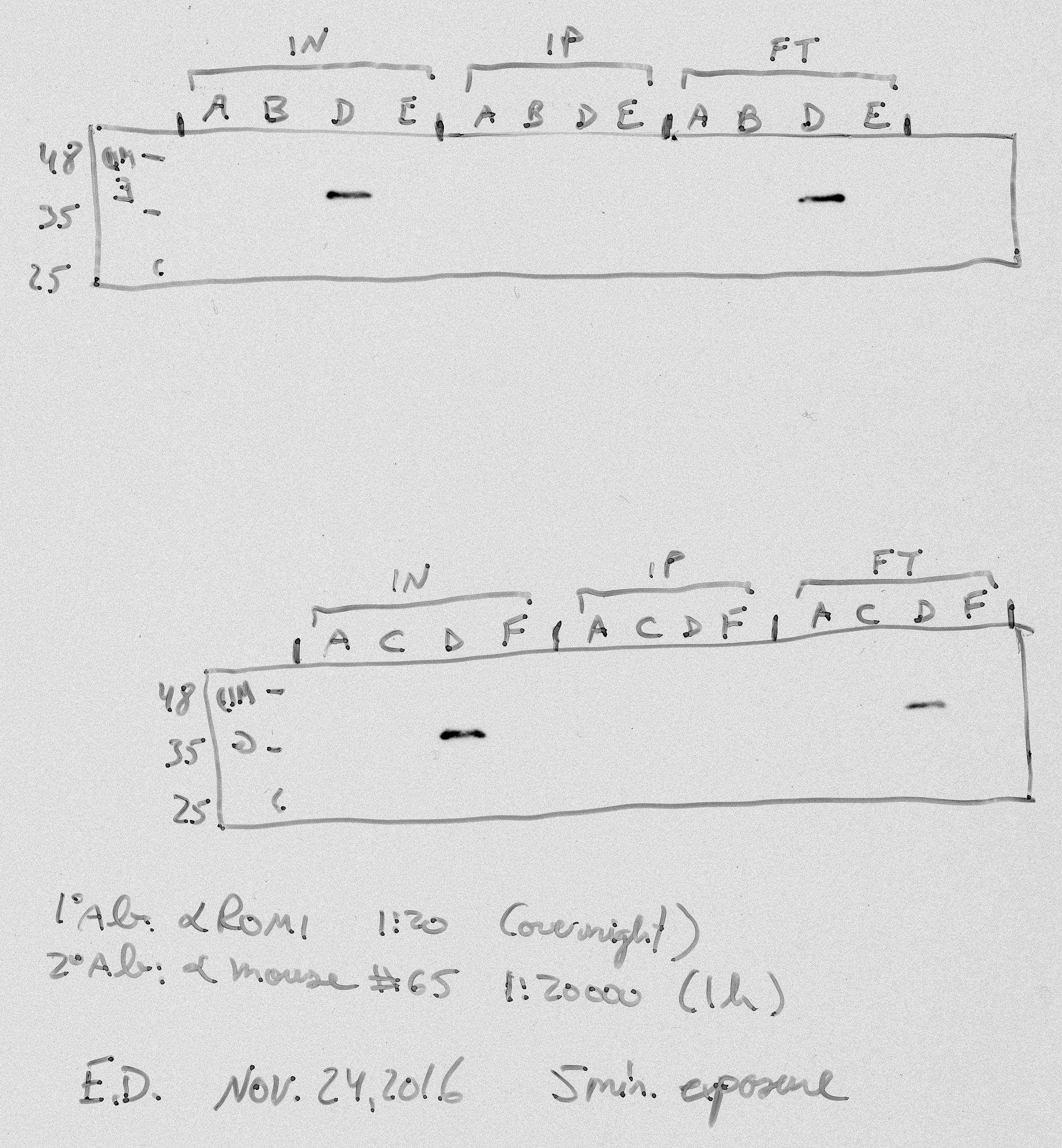

Supplement: Figure 7—source data 3. [file elife-107102-fig7-data3.zip › Figure 7A_2-source data 2/EML3 YWHA coIP 5min expo - ROM1.tif]

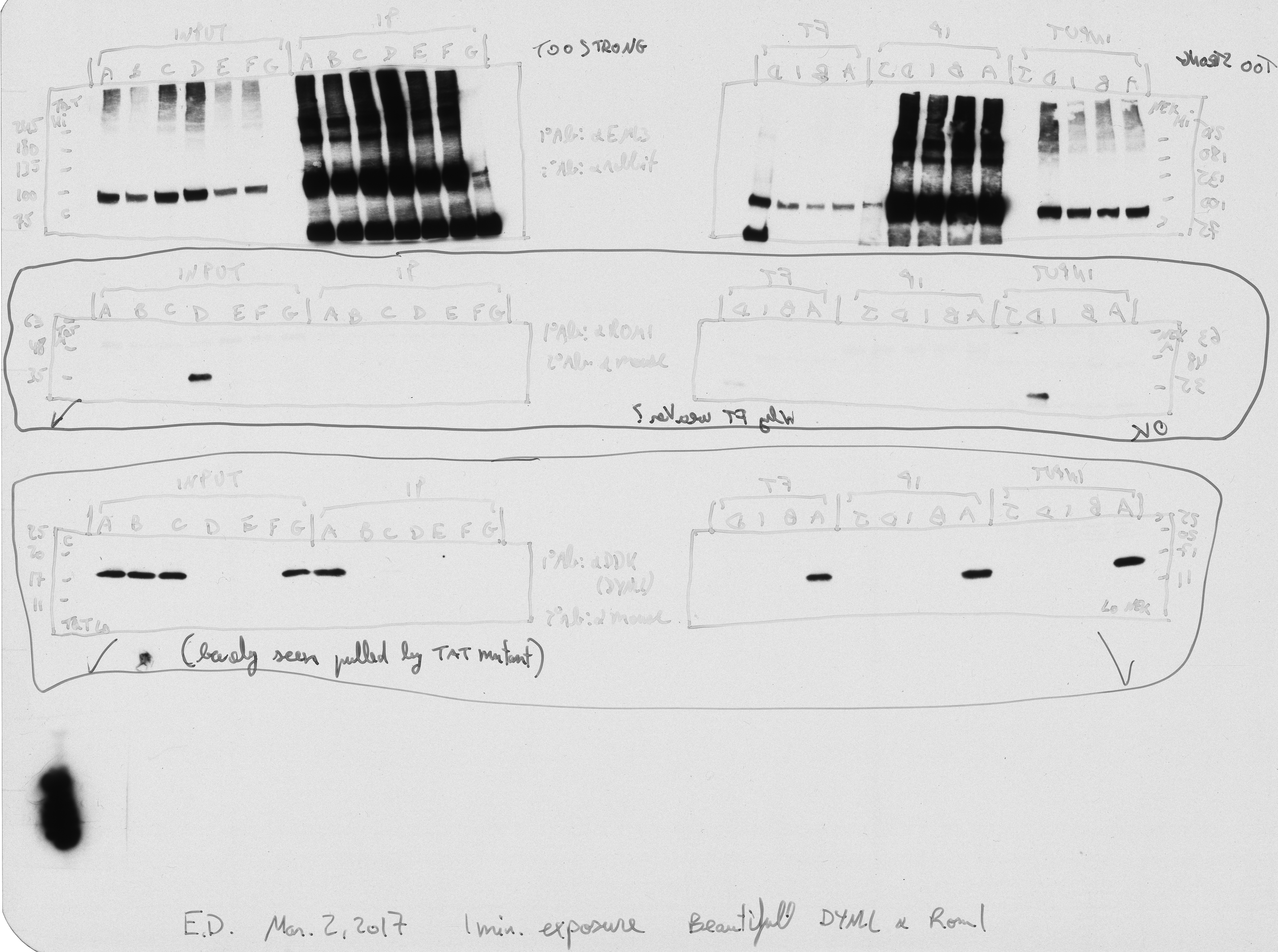

Supplement: Figure 7—source data 4. [file elife-107102-fig7-data4.zip › Figure 7B-source data 2/EML3 DYNLL coIP 1min expo - EML3 DYNLL1.tif]
