# Supplementary material for: The microtubule-binding protein EML3 is required for mammalian embryonic growth and cerebral cortical development, and Eml3 null mice are a model of cobblestone brain malformation
Source: eLife. 2026 Jul 9;14:RP107102. doi: 10.7554/eLife.107102 (PMC13349382; doi:10.7554/eLife.107102)
Supplement: Figure 7—source data 1. [file elife-107102-fig7-data1.zip › Figure 7-source data 1/Figure 7_Source Data 1.pptx]

## Slide 1
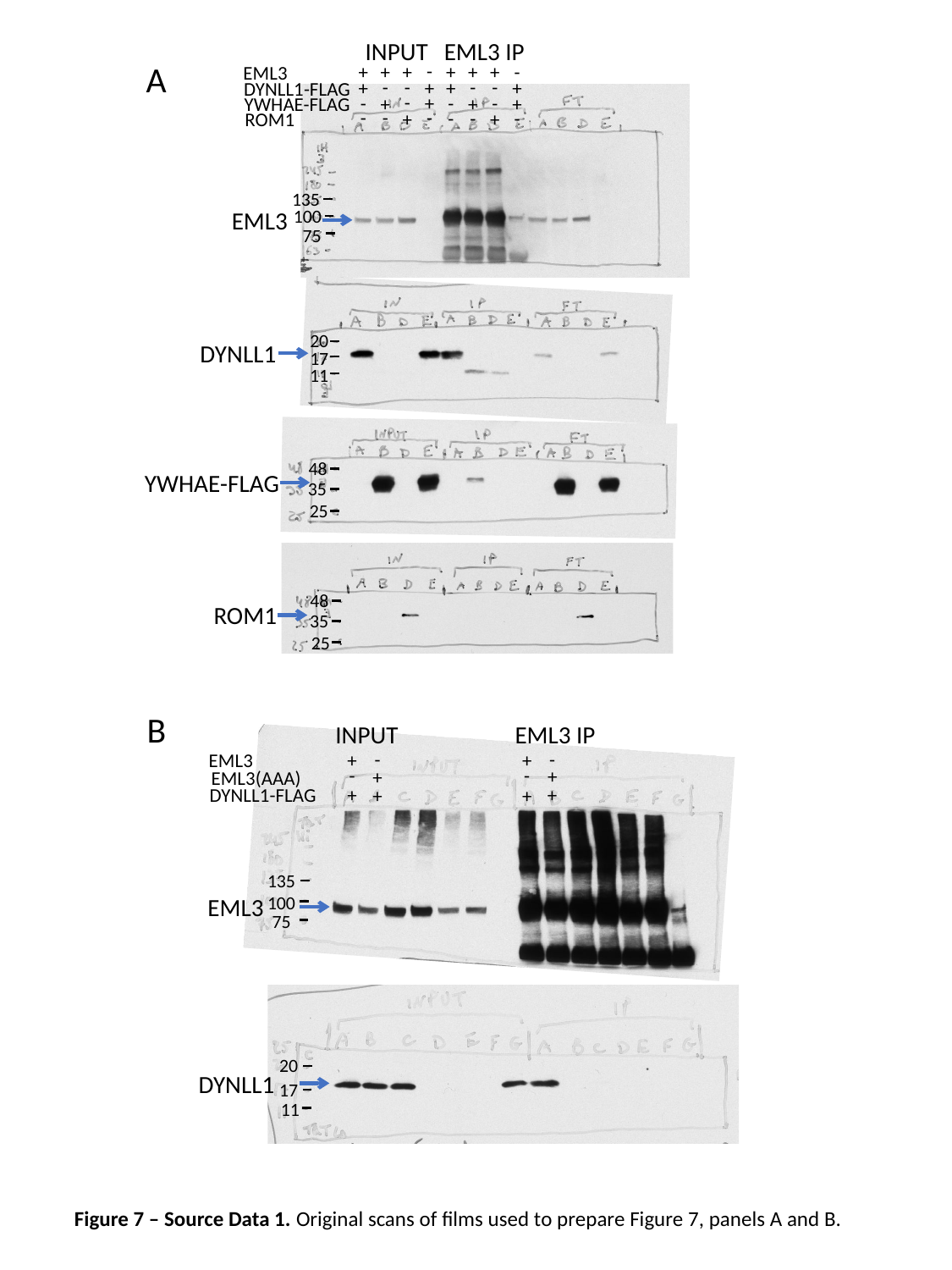

INPUT
EML3 IP
A
-
+
+
+
-
-
+
+
-
-
+
+
-
-
-
+
-
+
+
+
-
-
+
+
-
-
+
+
-
-
-
+
EML3
DYNLL1-FLAG
YWHAE-FLAG
ROM1
135
100
EML3
75
20
DYNLL1
17
11
48
YWHAE-FLAG
35
25
48
ROM1
35
25
B
INPUT
EML3 IP
-
-
+
+
EML3
-
-
+
+
EML3(AAA)
DYNLL1-FLAG
+
+
+
+
135
100
EML3
75
20
DYNLL1
17
11
Figure 7 – Source Data 1. Original scans of films used to prepare Figure 7, panels A and B.
