# Supplementary material for: The microtubule-binding protein EML3 is required for mammalian embryonic growth and cerebral cortical development, and Eml3 null mice are a model of cobblestone brain malformation
Source: eLife. 2026 Jul 9;14:RP107102. doi: 10.7554/eLife.107102 (PMC13349382; doi:10.7554/eLife.107102)
Supplement: Peer review history

# Editor report: eLife Assessment

**DOI:** 10.7554/eLife.107102.3.sa0

**Authors:** Ivan Velasco

**Affiliations:** Universidad Nacional Autónoma de México Mexico

**Keywords:** Convincing, Solid, Important

This **important** study is the first characterization of the phenotype caused by a lack of Eml3 expression in mice. Mutant animals present a disrupted pial basement membrane, leading to focal extrusions from the cerebral cortex, called ectopias. The methodology is **convincing** and the conclusions are **solid**, although further investigations on the molecular and cellular mechanisms are required to improve the manuscript. This work would be of interest to neural development biologists and human geneticists working on brain disorders.

# Referee report: Reviewer #2 (Public review)

**DOI:** 10.7554/eLife.107102.3.sa1

Summary:

In this manuscript, the authors investigate the role of the microtubule-binding protein EML3 during cortical development through the generation and characterization of an Eml3 mouse mutant. The authors focus mainly on the effects of EML3 loss on brain development, although Eml3 mouse mutants also present with developmental delay and growth restriction, and die perinatally due to respiratory distress caused by delayed maturation of the lungs. The main finding in the developing cortex is the presence of focal neuronal ectopias, which contain neurons from all cortical layers, as revealed by immunostaining. The authors use electron microscopy to show that ectopias seem to be caused by disruption to the pial basement membrane at early stages of development, which allows neurons to breach through it. To find a functional link between EML3 and the observed phenotype, studies are conducted that demonstrate expression of EML3 in radial glia cells and mesenchymal cells, both cell types involved in the formation and maintenance of the pial basement membrane. Furthermore, interaction partners for EML3 are identified through coIP-MS analysis, including tubulin beta-3, 14-3-3 proteins and cytoplasmic dynein light chain. However, mice carrying a mutant EML3 allele engineered to abolish the interaction between EML3 and cytoplasmic dynein light chain do not recapitulate any of the symptoms of complete EML3 loss.

Strengths:

The manuscript offers several important strengths that contribute significantly to the field. This study presents the first characterization of Eml3 knockout animals, providing novel insights into the role of Eml3 in vivo. Information on Eml3 function so far was restricted to cell culture data, so the results in this manuscript start to fill an important gap in our knowledge about this microtubule-binding protein. The experimental approach is carefully designed, with appropriate controls that ensure the reliability of the data. Moreover, the authors have addressed a key challenge in the analysis, namely the developmental delay of the knockout animals. By implementing a strategy to match developmental stages between wild-type and knockout groups, they allow for meaningful and valid comparisons between the two genotypes. Importantly, the authors have successfully generated three different Eml3 mutant mouse lines (knockout, floxed and with disrupted binding to cytoplasmic dynein light chain), which are very valuable tools for the broader scientific community to further study the roles of this gene in development and disease in the future.

Weaknesses:

While the manuscript presents valuable data, there are also several weaknesses that limit the overall impact of the study. Most notably, there is no clear mechanistic link established between the loss of Eml3 function and the observed phenotype, leaving the biological significance of the findings somewhat speculative, as it is not straightforward how a microtubule-associated protein can have an impact on the stability of the pial basement membrane. In this respect, but also in general for the whole manuscript, there seems to be a considerable amount of experimental work that has been conducted but is not presented, possibly due to the negative nature of the results. Additionally, the phenotype reported appears to be dependent on the genetic background, as it is absent in the CD1 strain. This observation raises concerns as to how robust the results are and how much they can be generalized to other mouse strains, but, more importantly, to humans.

# Referee report: Reviewer #3 (Public review)

**DOI:** 10.7554/eLife.107102.3.sa2

Summary:

This work aims to understand the role of Echinoderm Microtubule-associated Protein-like 3 (EML3) on embryogenesis and neocortical development. Importantly, this work shows that depletion of EML3 cause focal neuronal ectopias by disrupting the structural integrity of the pial basement membrane, describing a new model of cobblestone brain malformation. Another member of the EML family, EML1, has been already shown to trigger neuronal migration disorders, particularly subcortical band heterotopia by affecting cell polarity. The results presented here point to a different mechanism of action. The authors show that EML3 is expressed in radial glia cells and mesenchymal cells in the pial region and upon EML3 depletion (i.e., Eml3 mutant mice) the pial basement membrane is structurally damaged allowing migrating neuroblasts to ectopically migrate through. Answering, in this case, that the weakening of the pial basement membrane is a prerequisite of focal neuronal ectopias. The authors provide a meticulous characterization of the Eml3 mutant mice, strengthening the conclusions of the results.

Strengths:

The authors provide a very detailed analysis of the defects observed in Eml3 mutant mice, by providing not only results by inferred day of conception but by classifying embryos by their number of somite pairs.

Weaknesses:

Most of the weaknesses originally raised by the reviewer had been addressed.

# Author comment: Author response

**DOI:** 10.7554/eLife.107102.3.sa3

**Authors:** Isabelle Carrier, Eduardo Diez, Valerio E Piscopo, Susanne Bechstedt, Hans van Bokhoven, Myriam Srour, Albert M Berghuis, Stefano Stifani, Yojiro Yamanaka, Roderick McInnes

**Affiliations:** Lady Davis Research Institute Montreal Canada; Lady Davis Research Institute Montreal Canada; Montreal Neurological Institute and Hospital Montreal Canada; McGill University Montréal Canada; Radboud University Medical Centre Nijmegen Netherlands; Montreal Children Hospital, MUHC-RI, Montreal, Quebec, Canada. Montreal Canada; McGill University Montreal Canada; McGill University Montreal Canada; Rosalind and Morris Goodman Cancer Institute, Department of Human Genetics, McGill University Montreal Canada; McGill University Montreal Canada

The following is the authors’ response to the original reviews

The following revisions have been made to address most of the publicly available suggestions made by the Reviewers.

We have also corrected formatting issues in two figure panels:

Fig.1B: embryo ages added over placenta images.

Fig. 4D: fixed a truncated label.

**Reviewer #1 (Public review):**

The study would benefit from clearer evidence and additional experiments that would help to establish the molecular and cellular mechanisms underlying the brain phenotype, the central topic of the work.

We agree that additional experiments are necessary to elucidate the mechanism(s) by which EML3 deficiency causes the observed developmental phenotypes. However, as no further experimentation is possible due to the closure of our laboratory, we are committed to sharing available materials including custom antibodies and cryopreserved sperm from our mouse lines. We include previously generated experimental data not presented in the original submission. While these additional data do not reveal the mechanisms, we believe that sharing hypotheses that were experimentally ruled out will benefit the scientific community.

M&M: we have added a section listing several tissue-specific *Eml3* KOs generated. All of the generated cKO mice were indistinguishable from *Eml3^wt^* controls.

Supp. Fig. 2 with staining for major PBM components has been added. We have included antibody information to M&M.

**Reviewer #2 (Public review):**

(1) While the manuscript presents valuable data, there are also several weaknesses that limit the overall impact of the study. Most notably, there is no clear mechanistic link established between the loss of Eml3 function and the observed phenotype, leaving the biological significance of the findings somewhat speculative, as it is not straightforward how a microtubule-associated protein can have an impact on the stability of the pial basement membrane. In this respect, but also in general for the whole manuscript, there seems to be a considerable amount of experimental work that has been conducted but is not presented, possibly due to the negative nature of the results. At least some of those results could be shown, particularly (but not only) the stainings for the composition of the ECM components.

We agree that additional experiments are necessary to elucidate the mechanisms at play. While we cannot conduct further experiments, we provide additional existing data, including a new Supp. Fig. 2 showing ECM component staining. As this reviewer rightly anticipated, these results might not clarify the mechanism but sharing the hypotheses that were already experimentally tested will be helpful.

(2) Additionally, the phenotype reported appears to be dependent on the genetic background, as it is absent in the CD1 strain. This observation raises concerns as to how robust the results are and how much they can be generalized to other mouse strains, but, more importantly, to humans.

Indeed, we have determined that genetic background greatly influences the manifestation of developmental defects caused by absence or mutation of the EML3 protein in mice. Modifier genes appear to play a significant role in phenotypic expression. In humans, the presence or absence of such modifiers may result in a broad spectrum of outcomes from no clinical relevance, as seen in CD1 mice, to potential intrauterine mortality. We agree that this underscores the challenge of translating mouse model findings to human implications. Future studies could include a search for *EML3* non-coding regulatory mutations and expanded analysis of neuronal development defects, such as COB, as well as cases of intrauterine growth restriction (IUGR).

(3) There is no data included in the manuscript about the generation and analysis of the Eml3AAA/AAA mouse line. This is an important omission, especially as no details on the validation or phenotypic characterization of this additional mouse line are provided. Including these elements would greatly strengthen the rigor and interpretability of the work, especially if that mouse line is to be shared with the scientific community.

We acknowledge this oversight and have added a Materials and Methods section describing the generation of *Eml3* TQT86AAA mice. Validation of the *Eml3* TQT86AAA mice included showing absence of EML3-DYNLL binding in our co-IP MS data in Table 3. We state that the validated *Eml3* TQT86AAA mice were phenotypically indistinguishable from *Eml3^wt^* control mice.

**Reviewer #3 (Public review):**

(1) Besides the data provided in the figures, the authors report a significant amount of experiments/results as "Data not shown". Negative data is still important data to report, and the authors may want to choose some crucial "not shown data" to report in the manuscript.

We have incorporated key datasets previously omitted, with priority given to those specifically requested by Reviewer #2.

(2) Results in Figure 3A apparently contradict results in 3B. A better explanation of the results should improve understanding of the data. Even though the conclusion that the "onset and progression of neurogenesis is normal in Eml3 null mice" seems logical based on the data, the final numbers are not (Figure 3A) and this should be acknowledged, as well.

We provide further explanations for the data presented in figures 3A and 3B to better convey the fact that the two datasets are not contradicting. In essence, since *Eml3* null mice are developmentally delayed (as determined by the number of somites at a specific age, Fig. 1C), the milestones in neurogenesis are reached at a later age in *Eml3* null mice, thus at embryonic age E11.5 *Eml3* null mice have fewer TBR2-positive cells (Fig. 3A). However, *Eml3* null mice have reached the same neurogenesis milestones as their WT counterparts when they have the same number of somites (Fig. 3B).

Results section for Fig. 3: we provide additional explanations that reconcile the results shown in Fig. 3A and Fig. 3B.

(3) The authors should define which cell types are identified by SOX1 and PAX6.

We have defined the expression timing and cell identity marked by SOX1 and PAX6 in neural progenitors during cortical development.
